# Integrating Tumor Stroma Biomarkers With Clinical Indicators for Colon Cancer Survival Stratification

**DOI:** 10.3389/fmed.2020.584747

**Published:** 2020-12-07

**Authors:** Yong Chen, Wenlong Wang, Bo Jiang, Lei Yao, Fada Xia, Xinying Li

**Affiliations:** Department of General Surgery, Xiangya Hospital, Central South University, Changsha, China

**Keywords:** colon cancer, microenvironment, tumor stroma, immune cells, prognosis stratification

## Abstract

The tumor stroma plays an important role in tumor progression and chemotherapeutic resistance; however, its role in colon cancer (CC) survival prognosis remains to be investigated. Here, we identified tumor stroma biomarkers and evaluated their role in CC prognosis stratification. Four independent datasets containing a total of 1,313 patients were included in this study and were divided into training and testing sets. Stromal scores calculated using the estimation of stromal and immune cells in malignant tumors using expression data (ESTIMATE) algorithm were used to assess the tumor stroma level. Kaplan-Meier curves and the log-rank test were used to identify relationships between stromal score and prognosis. Tumor stroma biomarkers were identified by cross-validation of multiple datasets and bioinformatics methods. Cox proportional hazards regression models were constructed using four prognosis factors (age, tumor stage, the ESTIMATE stromal score, and the biomarker stromal score) in different combinations for prognosis prediction and compared. Patients with high stromal scores had a lower overall survival rate (*p* = 0.00016), higher risk of recurrence (*p* < 0.0001), and higher probability of chemotherapeutic resistance (*p* < 0.0001) than those with low scores. We identified 16 tumor stroma biomarkers and generated a new prognosis indicator termed the biomarker stromal score (ranging from 0 to 16) based on their expression levels. Its addition to an age/tumor stage-based model significantly improved prognosis prediction accuracy. In conclusion, the tumor stromal score is significantly negatively associated with CC survival prognosis, and the new tumor stroma indicator can improve CC prognosis stratification.

## Introduction

Colorectal cancer is the world's fourth most deadly cancer, accounting for ~10% of global cancer-related deaths each year (1, 2). Risk stratification and prognosis prediction of patients with colorectal cancer mainly rely on the tumor, lymph node, metastasis (TNM) classification system of the American Joint Committee on Cancer (3). However, this system provides useful but incomplete prognostic information, and additional clinicopathological and molecular characteristics should be considered to improve its prediction accuracy, such as mutation status, immune score, stromal components, and the presence of microsatellite instability (4–8).

Malignant solid tumors like colon cancer (CC) consist of not only tumor cells but also the tumor microenvironment (TME), which includes infiltrating immune cells, tumor stroma components, and other normal epithelial cells (9). The tumor stroma and immune cells are increasingly thought to play important roles in CC progression and drug resistance (10, 11); however, the specific molecules involved and their mechanisms remain unclear, particularly for the tumor stroma. Pagès et al. (12) developed a new indicator, termed an “immunoscore,” which could effectively predict CC prognosis. It measures the density of CD3+ and CD8+ T-cell effectors within the tumor and its invasive margins to assess the levels of infiltrating immune cells. We hypothesized that adding an additional indicator based on the tumor stroma into the current classification system would further improve CC prognosis stratification.

Estimation of stromal and immune cells in malignant tumors using expression data (ESTIMATE) is a newly developed algorithm that assesses the levels of the tumor stroma and infiltrating immune cells using the transcriptional profiles of cancer tissues, by detecting the specific gene expression signatures of stromal and immune cells (13). This method has been applied to several cancers and has proved helpful for prognosis stratification (14, 15); however, it has not been applied to CC. Based on this method, the purpose of this study was to develop a new specific tumor stroma indicator to improve the risk stratification and prognosis prediction of patients with CC.

## Materials and Methods

### Data Preparation

Normalized gene expression matrices and matched clinical information for GSE39582 and GSE17538, which contain 556 and 232 patients with CC, respectively, were downloaded from the Gene Expression Omnibus database. These microarray datasets, both acquired on Affymetrix Human Genome U133 Plus 2.0 Arrays, were combined for further analysis by correcting batch effects using the ComBat method implemented in the “SVA” package. Normalized mRNA expression and protein/phosphorylation expression matrices and matched clinical information from a dataset containing 106 patients with CC were obtained from the cBioPortal database (http://www.cbioportal.org/). TCGA project-COAD level 3 gene expression and micro (mi)RNA expression matrices, normalized by fragments per kilobase of exon per million reads mapped fragments (FPKM) and reads per million mapped reads (RPM), respectively, and a corresponding DNA methylation beta matrix were downloaded using the R package “TCGAbiolinks.” Inclusion criteria for patients were: (1) complete information regarding survival status and time; and (2) a follow-up time ≥1 month. Human reference genome annotation data (version: GRCh38.p13) and human binding motif data (version: GRCh38.p13) were downloaded from the Ensembl BioMart database (https://useast.ensembl.org/index.html) to predict transcription factors (TFs) regulating target genes.

### Correlations Between the ESTIMATE Stromal Score and Clinical Prognosis

The ESTIMATE algorithm was applied to calculate the stromal score of each CC patient using gene expression profiles. To identify the most significant stromal score threshold for patient grouping, we used the method “maximally selected rank statistics” in the R package “maxstat” (16). Patients were divided into high and low stromal score groups according to the threshold value. Then, Kaplan-Meier (KM) analysis and a log-rank test were used to identify survival differences between the high and low stromal score groups in the training set, and validation was performed using the testing sets. Moreover, we performed Wilcoxon rank-sum and/or Kruskal-Wallis tests to identify relationships between the ESTIMATE stromal score and clinical features, including T, N, and M pathological results and the tumor stage.

### Correlations Between the ESTIMATE Stromal Score and Chemotherapy Resistance

A subset of 540 patients from GSE39582 with information regarding adjuvant chemotherapy was divided into three groups based on their treatment regimens and stromal scores: patients who were not treated with chemotherapy, patients with low stromal scores who were treated with chemotherapy, and patients with high stromal scores who were treated with chemotherapy. Then, we performed Wilcoxon rank-sum and Kruskal-Wallis tests to identify differences in the stromal score distribution between the three groups. KM analyses and log-rank tests were used to identify survival differences.

### Identification of Specific Differentially Expressed Genes (SDEGs)

To identify SDEGs in the high stromal score group vs. the low stromal score group, we analyzed differences between the groups in three independent datasets (the training set and the testing sets). The R package “limma” was used to identify differentially expressed genes (DEGs), based on thresholds of log fold change >1 and adjusted *p* (adj*P*) <0.05. Then, we performed overlap analysis of the top 30 DEGs from each dataset to identify SDEGs that were significantly increased in the high stromal score group compared to the low stromal score group.

### Identification of Clinically Significant Modules

We conducted weighted co-expression network analysis (WGCNA) to identify modules most relevant to the tumor stroma and characterize the correlation patterns among module genes using the R package “WGCNA.” The mRNA weighted co-expression network was constructed using the mRNA expression profile in the training set and the top 10,000 variable genes measured by median absolute deviation. The “WGCNA” package function pickSoftThreshold was used to select an appropriate soft-thresholding power value, which was applied to construct a scale-free topology matrix. Parameters used to construct the co-expression gene modules were as follows: a deepSplit of 2, a minModuleSize of 30, a maxBlockSize of 20,000, and merging of highly similar modules when the module eigengene height in the clustering was <0.25. Finally, we related the modules to clinical features to identify the module whose genes were most relevant to the stromal score.

### Module Preservation Analysis and Functional Annotation

To examine the stability of the identified stroma-related module, we performed module preservation analysis using the function modulePreservation (17) in the “WGCNA” package and the two mRNA expression profiles in the testing sets, with the parameter nPermutation set to 200. The preservation Zsummary (Z) was used to estimate module preservation between different datasets, with Z > 10, 5 < Z ≤ 10, and Z ≤ 5 indicating high, median, and low preservation, respectively. Then, to explore the biological functions of the genes in the stroma-related module, we performed gene ontology (GO) and Kyoto Encyclopedia of Genes and Genomes (KEGG) pathway enrichment analyses using the R package “clusterProfiler.” Adj*P* < 0.01 was considered statistically significant.

### Hub Gene Identification

Hub genes within modules are genes that have a high degree of connectivity in the associated interaction network and play important roles in related clinical features. To identify hub genes in the stroma-related module, we first constructed a protein-protein interaction (PPI) network containing all genes in the module using the online database STRING (https://string-db.org/). Then, we imported the PPI network into Cytoscape (version 3.71) to calculate the degree of each node. Candidate hub genes had degrees >90. We also performed overlap analysis between candidate hub genes and the three DEG sets to further filter the hub genes.

### Biomarker Identification

In this study, tumor stroma biomarkers were defined as closely related to the stromal score and significantly negatively correlated with survival prognosis. All identified SDEGs and hub genes were initially selected as candidate biomarkers. We first conducted *t*-tests to further validate the expression differences of these genes between the high and low stromal score groups at the protein level using the protein/phosphorylation expression matrix. Protein features containing >30% missing values were excluded prior to the *t*-test. The criterion for filtering was *p* < 0.05. Next, we conducted Pearson correlation analyses using the mRNA expression profile from the training set to determine the relationships between candidate biomarkers and the stromal score. The criteria for screening were *p* < 0.01 and *r* > 0.5. The results were verified by the same method using the testing sets.

### Correlations Between Biomarkers and Prognosis

We divided patients in the training set into high and low expression groups according to the optimal cutoff of each biomarker's mRNA expression, as determined by the R package “maxstat.” Then, we performed KM analysis and log-rank tests to determine survival differences between the two groups based on each biomarker. Statistical significance was defined as *p* < 0.05. We validated the results in the same manner using the testing sets. Biomarkers that produced statistically significant differences in both the training set and the testing sets were retained for further analysis.

### Construction of the Prognosis Model

In addition to the stromal score calculated by ESTIMATE, we created another new indicator for risk stratification, termed the biomarker stromal score, a cumulative measure of the number of biomarkers that were significantly higher in each patient. We divided the patients into low-, median-, and high-risk groups based on their biomarker stromal scores using the R package “maxstat,” then performed KM analysis and log-rank tests to determine survival differences between the three groups using survival information from all patients in the training set and the testing sets. Moreover, to estimate and compare the stratification ability of each prognosis feature, we performed time-dependent receiver operating characteristic (ROC; 3-year and 5-year) analysis with 1,000× bootstrap resampling for each feature (age, pathology T, pathology N, pathology M, tumor stage, ESTIMATE stromal score, and biomarker stromal score) separately. Finally, we performed multivariate regression analyses to construct three multivariable Cox proportional hazards models using the prognosis features age, tumor stage, ESTIMATE stromal score, and biomarker stromal score in different combinations. Two evaluation methods [time-dependent ROC curves (area under the curve (AUC) and the concordance index (C-index)] were used to measure the prediction accuracy of each prognosis model with 1,000× bootstrap resampling, and their performance was compared using the *p*-value of the likelihood ratio. In addition, to use the prediction model clinically, a nomogram was developed to predict the 1–5-year survival rates of patients with CC, and calibration curves were used to test its performance.

### Construction of the Direct Regulatory Network

To explore potential regulatory mechanisms of the biomarkers, we examined their methylation status, TFs, and competing endogenous RNA (ceRNA) networks. We analyzed methylation differences between the high and low stromal score groups using the R packages “ChAMP” and a methylation beta matrix containing 281 patients to detect CpG sites with significant changes in methylation. The thresholds for statistical significance were adj*P* < 0.05 and deltaBeta < -0.05.

We used the human reference genome annotation dataset and human binding motif dataset, which uses the position weight matrix method to predict potential TF binding sites, to predict TFs that interact with target gene promoters. Binding sites with scores <0 were filtered out of the binding motif dataset, and the promoter region of a gene was defined as the region between 1,000 bp upstream and 200 bp downstream of the transcriptional start site in the genome annotation dataset. We further filtered the TFs according to their differential expression in the high and low stromal score groups using the protein/phosphorylation expression matrix. Moreover, to improve the confidence of the TF assignments, we performed Pearson correlation analysis to identify associations between TFs and target genes using a subset from dataset100 containing 96 patients with both mRNA expression and protein/phosphorylation expression profiles, with thresholds of *p* < 0.05 and *r* > 0.3.

Finally, given the positive regulatory associations between long non-coding (lnc)RNAs and mRNAs in ceRNA networks, we first performed Pearson correlation analysis to examine associations between the expression of lncRNAs and the biomarkers using a dataset containing 453 patients with both lncRNA and mRNA expression profiles. The criteria for filtering lncRNAs were *r* > 0.65 and *p* < 0.01. We predicted direct miRNA-mRNA interactions using the online database StarBase (http://starbase.sysu.edu.cn/). For inclusion, interactions needed to be validated at least once by cross-linking immunoprecipitation(CLIP), and predicted by at least three of the PITA, RNA22, miRmap, miroT, miRanda, PicTar, and TargetScan databases. Direct lncRNA-miRNA interactions were predicted using the starBase miRanda tool. For inclusion, interactions needed to be validated at least once by CLIP. Then, we merged the lncRNA-miRNA and miRNA-mRNA networks to generate the direct lncRNA-miRNA-mRNA regulatory network. Finally, we performed KM analysis and log-rank tests to identify survival differences based on the expression levels of lncRNAs and miRNAs in the ceRNA network, using lncRNA and miRNA expression matrices containing 428 and 413 patients, respectively. Statistically significant (*p* < 0.05) lncRNAs and miRNAs were retained. The network was constructed and visualized using Cytoscape.

### Statistical Analyses

All statistical analyses in this study were completed in R version 3.6.3 (https://www.r-project.org/). Appropriate R packages were used for different analyses. For these, specific parameters used are listed in their respective sections, while default parameters are not listed. The threshold of statistical significance varied among different statistical analyses but was at least *p* < 0.05.

## Results

### Data Collection

**Figure 1:**
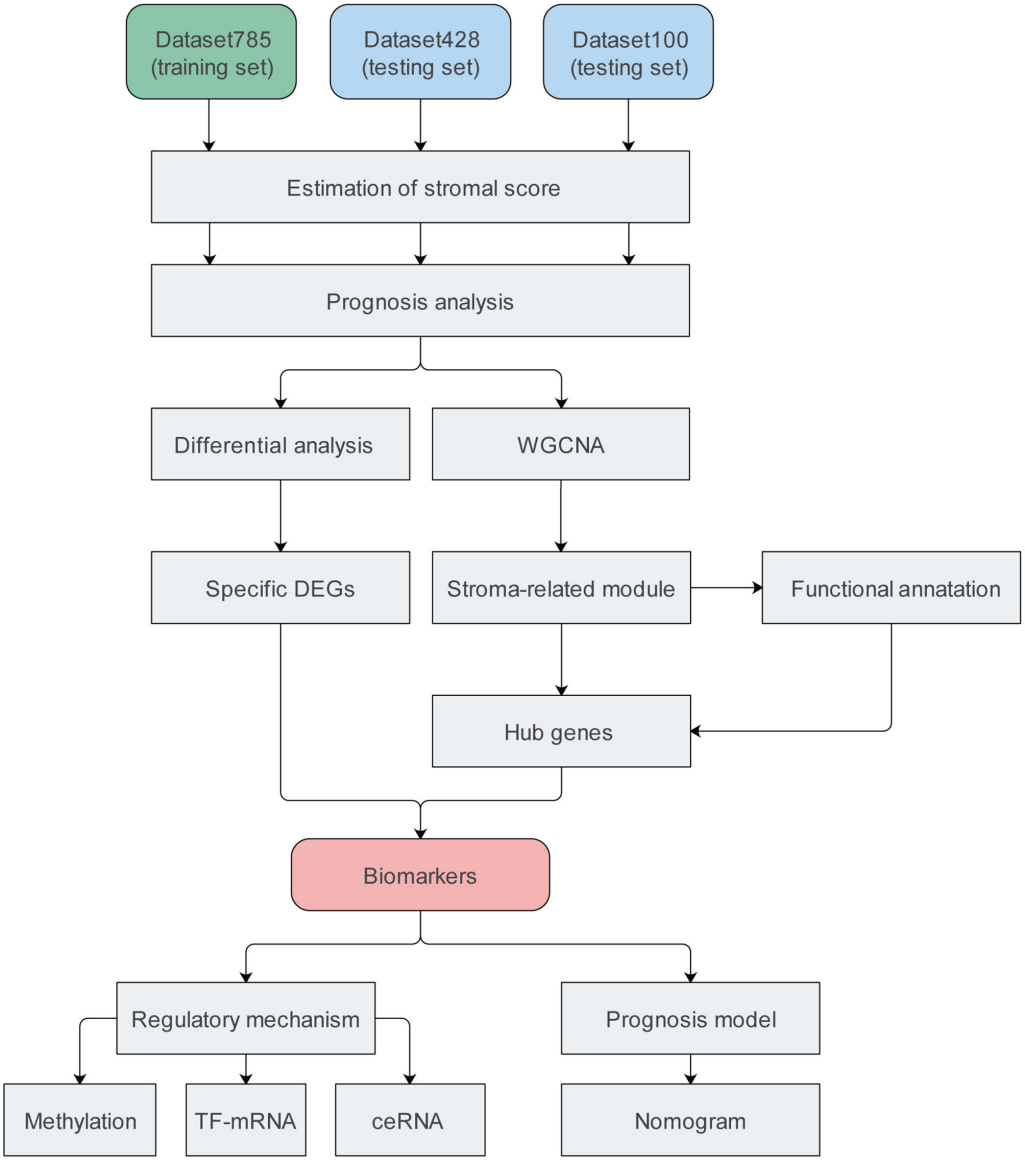
Flow chart of the study. DEG, differentially expressed gene; TF, transcription factor; ceRNA, competing endogenous RNA.

We included four datasets containing nine expression matrices and a total of 1,313 patients with primary CC. Different expression matrices in the same dataset shared the same patients; however, the number of patients in the matrices were not necessarily the same. The GSE39582 and GSE17538 datasets were combined into a training set with an mRNA expression matrix containing 785 patients, termed dataset785. This set was mainly used to mine data in our study. A dataset containing 100 patients was obtained from the cBioPortal database was termed dataset100. It consisted of mRNA and protein/phosphorylation expression matrices. Another dataset, obtained from The Cancer Genome Atlas (TCGA), containing 428 patients and mRNA, lncRNA, miRNA, and methylation expression matrices, was termed dataset428. Dataset100 and dataset428 were defined as testing datasets mainly used for verification and molecular mechanism analysis. Details regarding the datasets are provided in Supplementary Table 1, and the complete workflow of the study is displayed in Figure 1.

### Correlations Between the ESTIMATE Stromal Score and Clinical Prognosis

**Figure 2:**
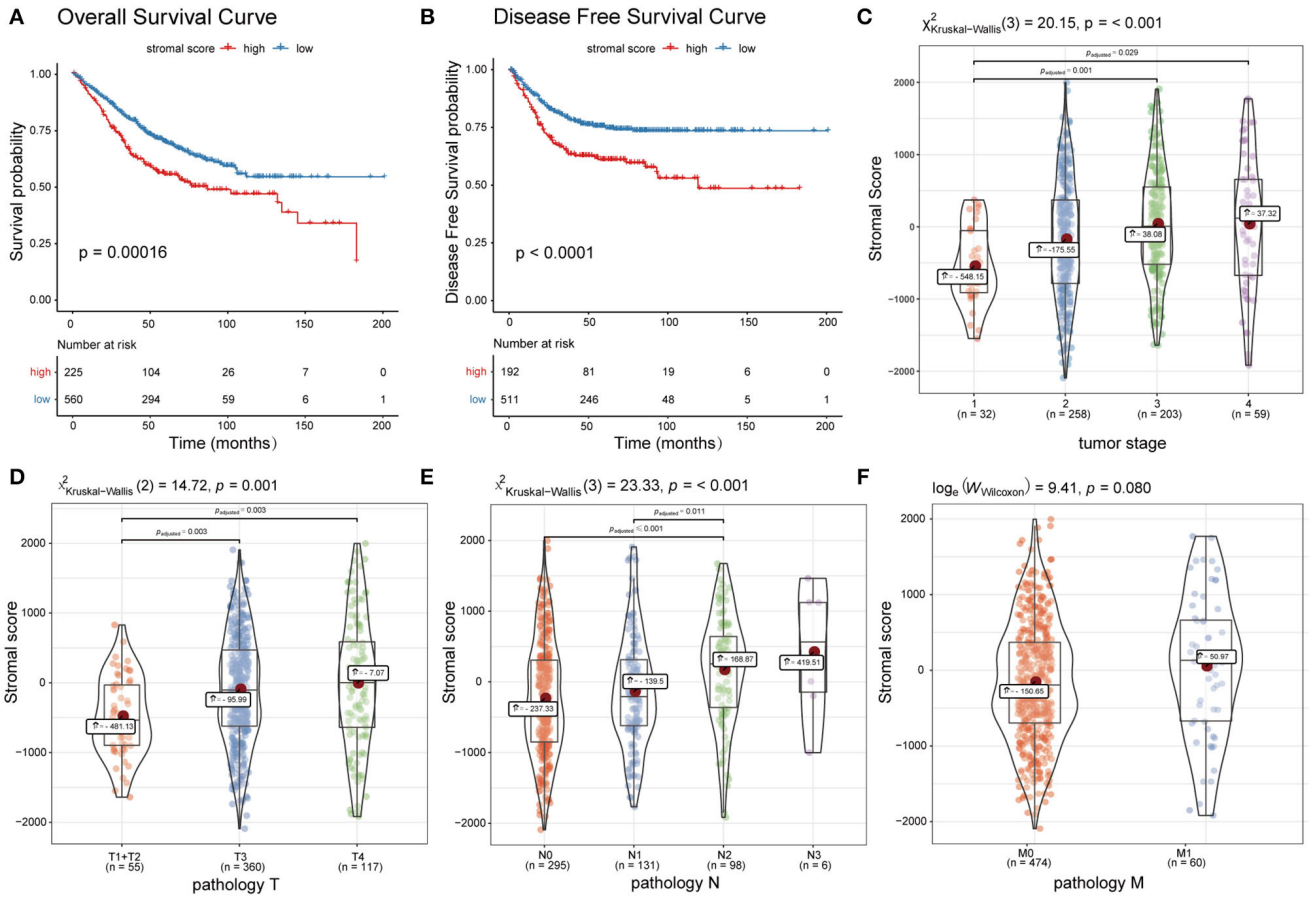
Association between the ESTIMATE stromal score and CC survival prognosis and clinical features. **(A)** Overall survival curves and **(B)** disease free survival curves of the high and low stromal score groups. **(C–F)** Boxplots of the ESTIMATE stromal scores of different groups with regards to T, N, and M pathology results and tumor stage.

Patients in dataset785 were divided into high and low stromal score groups based on the determined optimal cutoff. KM analysis and a log-rank test revealed that patients with low scores had significantly better overall survival (OS; *p* = 0.00016; Figure 2A) and disease-free survival (DFS; *p* < 0.0001; Figure 2B) than patients with high scores. These results were validated using dataset100 and/or dataset428 (Supplementary Figures 1A–C). Wilcoxon rank-sum and Kruskal-Wallis tests identified statistically significant relationships between the stromal score and clinical features, including the T, N, M pathology results and tumor stage (Figures 2C–F). The results were verified using dataset100 (Supplementary Figures 2A–D). Therefore, these results indicate that tumor stroma is closely associated with tumor progression and survival prognosis.

### Correlations Between the ESTIMATE Stromal Score and Chemotherapy Resistance

**Figure 3:**
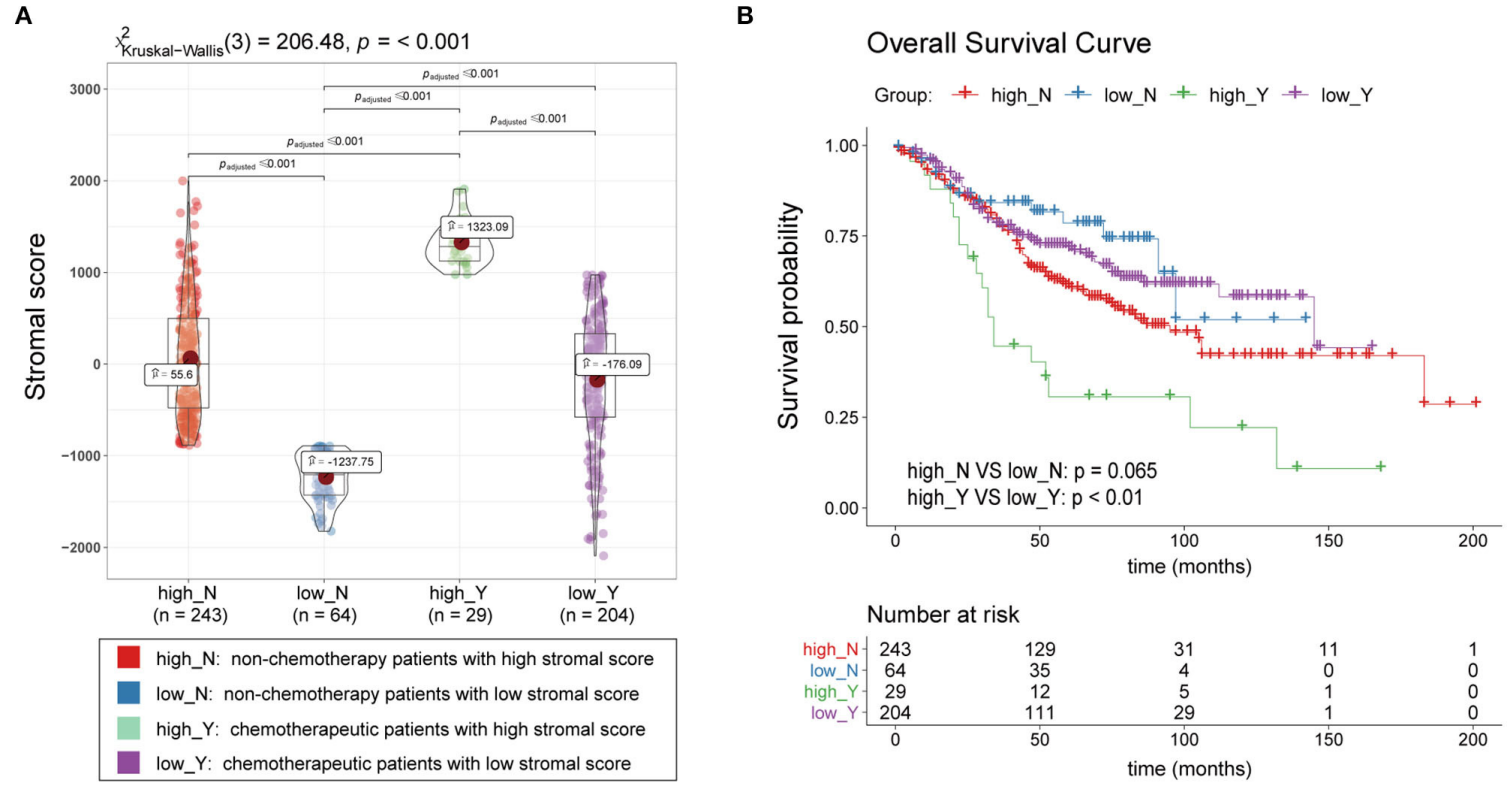
Association between the ESTIMATE stromal score and chemotherapeutic resistance. **(A)** Boxplots of the stromal scores and **(B)** survival curves of patients with high and low stromal scores who were treated with chemotherapy and patients who were not treated with chemotherapy.

A total of 540 patients with information regarding adjuvant chemotherapy were included and divided into four groups based on the ESTIMATE stromal score and adjuvant chemotherapy information. Wilcoxon rank-sum and Kruskal-Wallis tests showed the distribution of the ESTIMATE stromal score between groups were significantly different and the details were shown in Figure 3A. Patients treated with chemotherapy who had high stromal scores had a lower OS rate than those with low stromal scores and patients who were not treated with chemotherapy (*p* < 0.01); however, there was no significant difference in survival between chemotherapy patients who had low stromal scores and patients not treated with chemotherapy (Figure 3B). Therefore, our findings indicate that patients treated with chemotherapy who have high stromal scores are more vulnerable to the development of chemotherapeutic tolerance and have a poor survival prognosis.

### SDEG Identification

**Figure 4:**
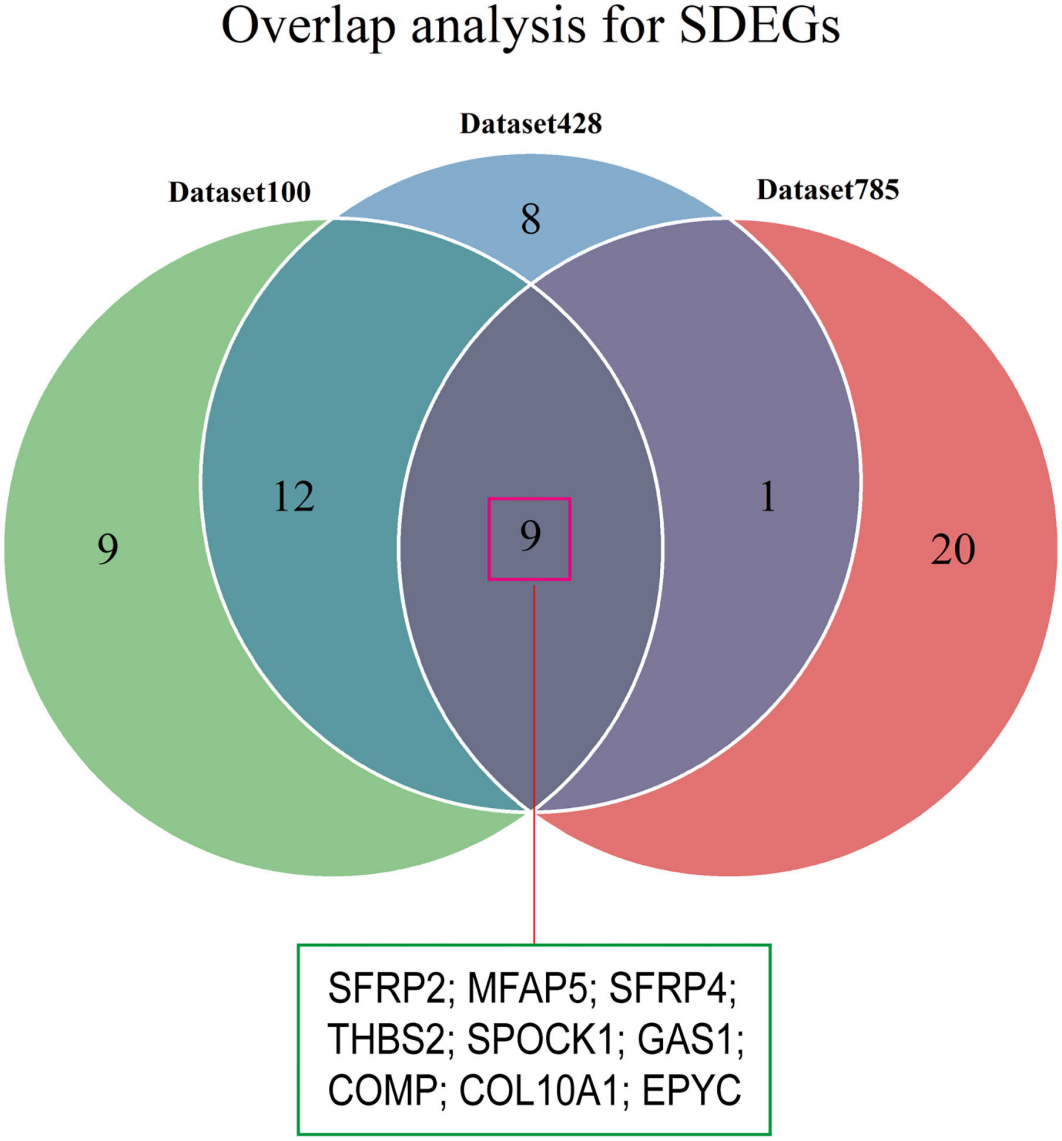
Identification of SDEGs. The Venn diagram shows the overlap between the top 30 DEGs from the three datasets.

We conducted differential analyses between the high and low stromal score groups using the mRNA expression profiles in dataset785, dataset100, and dataset428, and identified 246, 501, and 2,313 DEGs, respectively (Supplementary Figures 3A–C). Overlap analysis of the top 30 DEGs from each DEG set (based on the log fold change) produced nine SDEGs (Figure 4 and Table 1). Notably, among these nine SDEGs, gene SFRP2 had the biggest logFC. These SDEGs may play an important role in tumor stroma-induced promotion of tumor progression.

**Table 1:** Differential analysis statistics of the nine SDEGs.

**Gene**	**Dataset785**		**Dataset428**		**Dataset100**	
	**logFC**	**adj. *P*.Val**	**logFC**	**adj. *P*.Val**	**logFC**	**adj. *P*.Val**
SFRP2	3.33	5.2E-114	4.14	7.2E-34	3.25	2.2E-06
COL10A1	2.50	2.3E-85	3.47	6.0E-28	2.40	4.6E-06
SFRP4	2.37	1.3E-93	3.32	1.2E-31	2.06	9.9E-05
THBS2	2.11	1.6E-82	2.88	4.0E-38	1.91	1.1E-06
SPOCK1	2.11	1.6E-104	2.98	2.3E-36	1.96	7.2E-07
MFAP5	1.98	5.4E-96	2.96	4.4E-37	1.89	5.5E-07
COMP	1.96	1.7E-79	3.66	1.4E-31	2.76	3.6E-05
EPYC	1.74	1.3E-49	3.65	5.0E-27	2.92	2.6E-05
GAS1	1.70	9.6E-115	3.13	7.1E-40	2.42	4.9E-07

*SDEGs, specific differentially expressed genes; logFC, log fold change; adj.P.Val, adjusted p-value*.

### Identification of Stroma-Related Modules Through WGCNA

**Figure 5:**
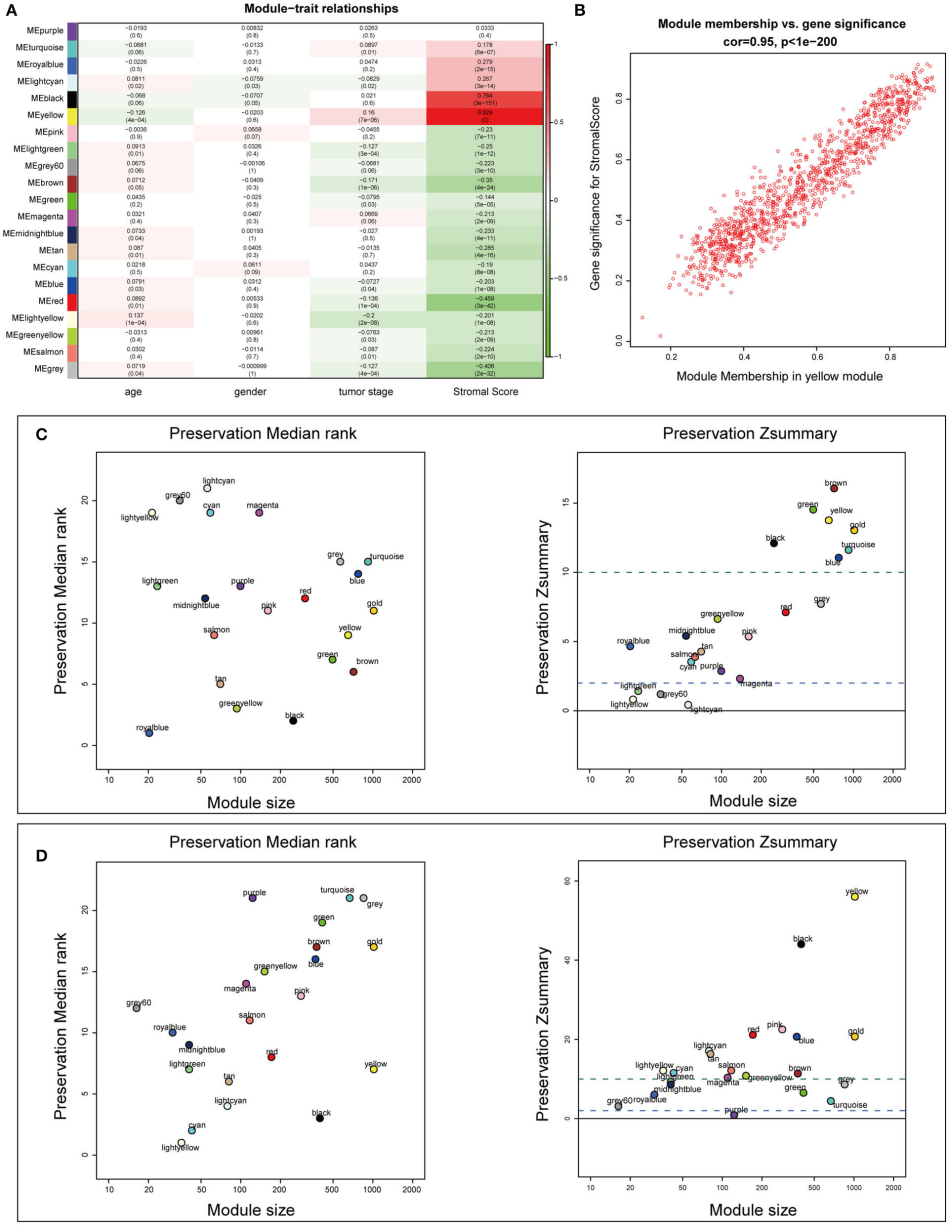
Identification of stroma-relevant mRNA modules and module preservation analysis. **(A)** Heatmap of module-trait relationships. **(B)** Scatter plot of correlations between gene module membership and gene significance in the yellow module. **(C,D)** Preservation medianRank and Zsummary graphs of the testing sets dataset100 and dataset428. Dashed blue and green lines show the thresholds Z = 2 and Z = 10, respectively.

To construct the mRNA co-expression network, we selected 6 as the appropriate sort-thresholding power value, which generated 21 mRNA modules (Supplementary Figures 4A–C). Association analysis between the modules and clinical features revealed that the yellow module (containing 1,173 genes) was most related to the stromal score (*r* = 0.929; *p* = 0; Figures 5A,B). This indicates that genes in the yellow module, particularly its hub genes, may play important roles in the tumor stroma-induced promotion of tumor progression and drug resistance.

### Module Preservation Analysis and Functional Annotation

**Figure 6:**
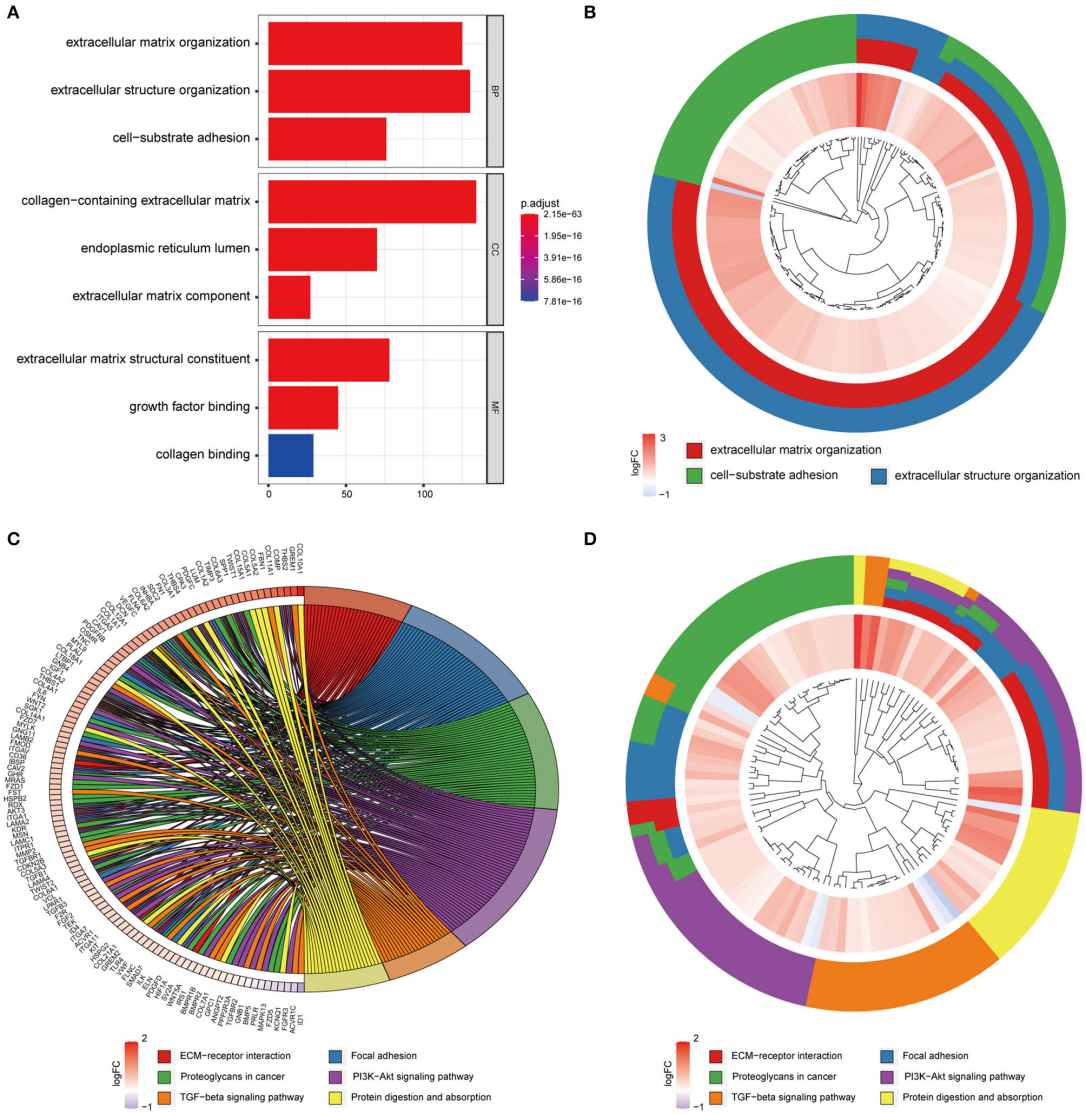
GO and KEGG enrichment analysis of genes in the yellow module. **(A)** Barplot of the top three most statistically significant GO terms in the BP, CC, and MF categories. **(B)** Circle diagram of the three most statistically significant BPs. **(C,D)** Circle diagram of the six most significant KEGG pathways.

To examine the stability of the stroma-related module (yellow) in the training set identified above, we performed module preservation analyses using the two testing sets (dataset428 and dataset100). As shown in Figures 5C,D, the horizontal dashed lines indicate the Zsummary (Z) thresholds for strong evidence of conservation (>10) and for low to moderate evidence of conservation (>2), so we can see the yellow module had good performance with Z > 10 in both dataset428 and dataset100, which means that genes in the yellow module have high consistency in the training set and testing sets. Moreover, to determine the functional involvement of the tumor stroma, the 1,173 genes in the yellow module were subjected to GO and KEGG pathway enrichment analyses. As shown in Figures 6A,B, enriched biological processes (BPs), molecular functions (MFs), and cellular components were all significantly focused on the extracellular matrix (ECM). Most of genes in the six most statistically significant signaling pathways were overexpressed in the high stromal score group, and the three pathways “ECM-receptor interactions,” “focal adhesions,” and “PI3K-Akt signaling pathway” shared a significant number of genes (Figures 6C,D). This indicates that these biological processes and signaling pathways are closely related to tumor stroma function.

### Identification of Hub Genes

**Figure 7:**
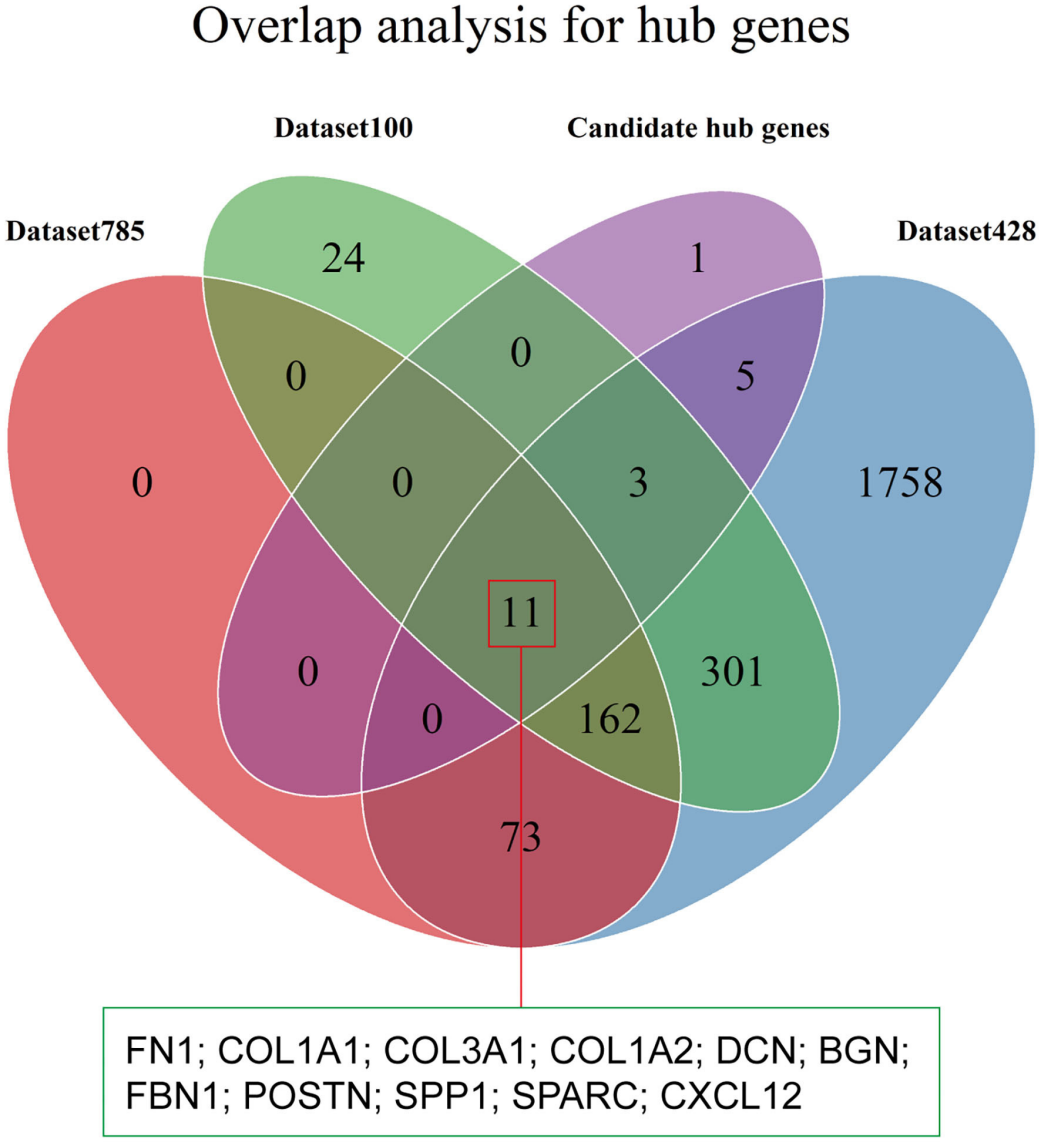
Identification of hub genes. The Venn diagram shows the overlap between the 20 candidate hub genes with degrees of connectivity >90 and the three DEG sets.

Hub genes were defined as genes with high degrees of connectivity in a PPI network of the yellow module. The interaction network contained 1,105 nodes and 8,927 edges, and node degrees ranged from 1 to 220 (Supplementary Figure 5). We selected 20 candidate hub genes based on a degree threshold of ≥90. Overlap analysis of candidate hub genes from the three DEG sets identified 11 hub genes (Figure 7 and Table 2). This means that these 11 hub genes may have important impacts on the function of tumor stroma.

**Table 2:** Statistics of the 11 hub genes in the yellow module.

**Genes**	**Degrees of connectivity**	**Dataset785**		**Dataset428**		**Dataset100**	
		**logFC**	**adj. *P*.Val**	**logFC**	**adj. *P*.Val**	**logFC**	**adj. *P*.Val**
FN1	220	1.24	4.1E-61	2.53	2.9E-31	1.74	1.2E-04
COL1A1	137	1.04	3.9E-64	2.29	1.2E-36	1.60	9.1E-04
COL3A1	119	1.29	2.4E-69	2.22	9.3E-41	1.33	1.6E-06
COL1A2	116	1.37	3.6E-62	2.19	1.4E-38	1.36	4.0E-05
DCN	105	1.07	2.1E-99	2.17	6.1E-36	1.22	5.8E-07
BGN	100	1.42	1.1E-87	2.16	2.5E-41	1.78	4.6E-06
FBN1	98	1.69	3.5E-113	2.21	1.4E-42	1.30	1.8E-07
POSTN	98	1.29	1.2E-76	2.56	1.2E-33	1.70	1.5E-07
SPP1	98	1.39	2.1E-42	3.03	2.8E-25	2.06	7.1E-05
SPARC	94	1.40	4.5E-91	1.83	2.2E-42	1.26	4.1E-08
CXCL12	91	1.43	2.7E-87	1.85	8.9E-40	1.54	4.7E-08

*Degrees of connectivity, edge counts of genes in the PPI network calculated by Cytoscape; logFC, log fold change; adj.P.Val, adjusted p-value*.

### Identification of Tumor Stroma Biomarkers

**Figure 8:**
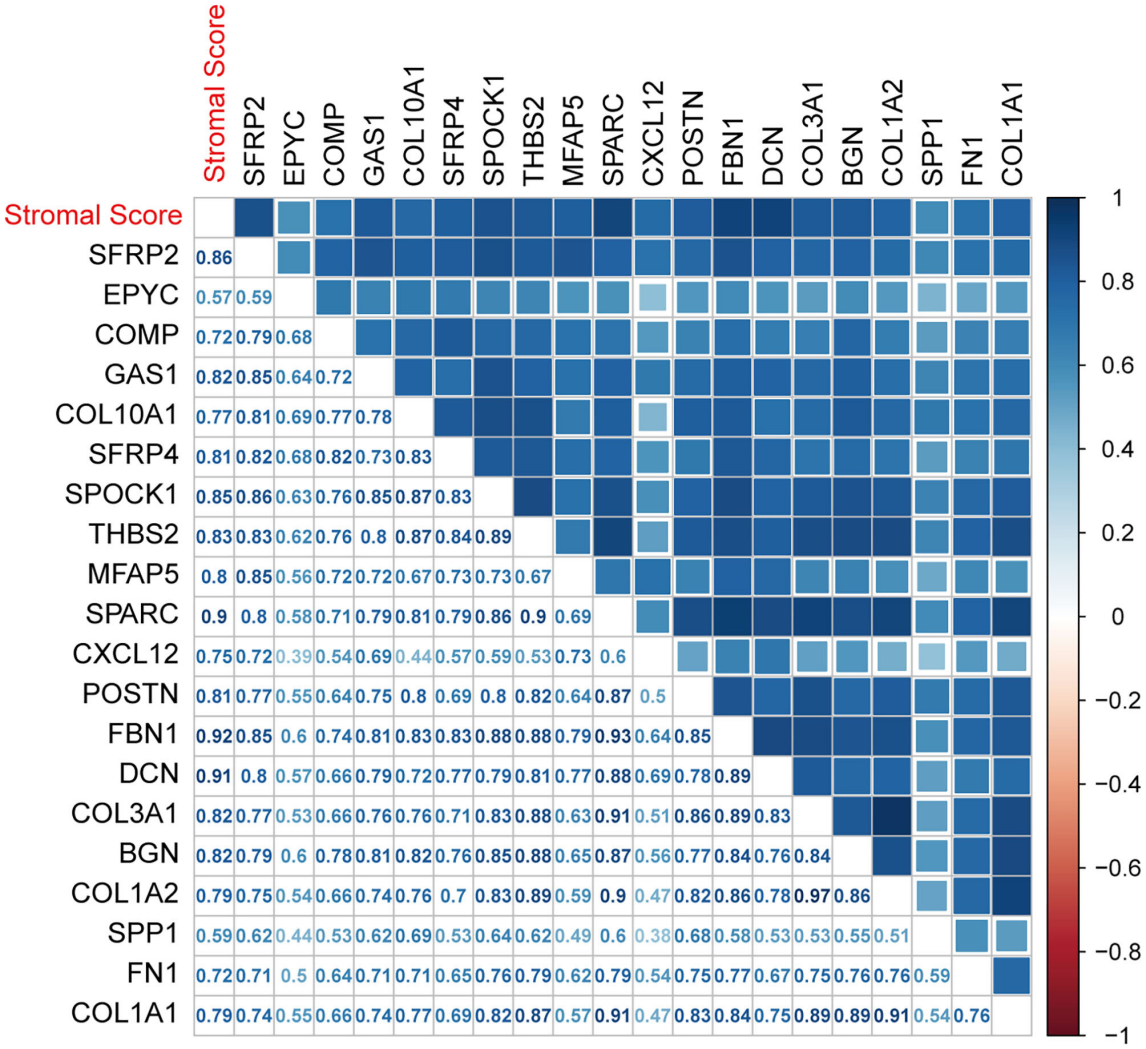
Associations between biomarkers and the ESTIMATE stromal score. The correlation plot was generated by Pearson correlation analysis.

The nine SDEGs and 11 hub genes were considered candidate tumor stroma biomarkers and were all significantly related to the stromal score based on thresholds of *r* > 0.5 and *p* < 0.01 (Figure 8). The results were verified using the two testing sets (Supplementary Figures 6A,B). *t*-tests using the protein expression matrix revealed that 16/20 genes were significantly overexpressed in the high stromal score group compared to the low stromal score group (Supplementary Table 2). Four genes [epiphycan (EPYC), growth arrest specific 1 (GAS1), SPARC (osteonectin), cwcv- and kazal-like domains proteoglycan 1 (SPOCK1), and secreted phosphoprotein 1 (SPP1)] were not included in the protein expression matrix; therefore, we are unable to determine whether they display differential protein expression. Given their significant differential mRNA expression, these four genes were retained, resulting in 20 candidate biomarkers for further analysis. Among these candidate biomarkers, four collagen family members (COL1A1, COL1A2, COL3A1, COL10A1) which are well-known to be closely related to the function of the stroma were contained, which also strongly supports the reliability of our results. However, due to the heterogeneity of the tumor microenvironment, even the same markers may play different roles in different cancers. Therefore, although some markers found in our study were closely related to the prognosis in CC, they may play different roles in other cancers.

### Correlations Between Tumor Stroma Biomarkers and Survival Prognosis

**Figure 9:**
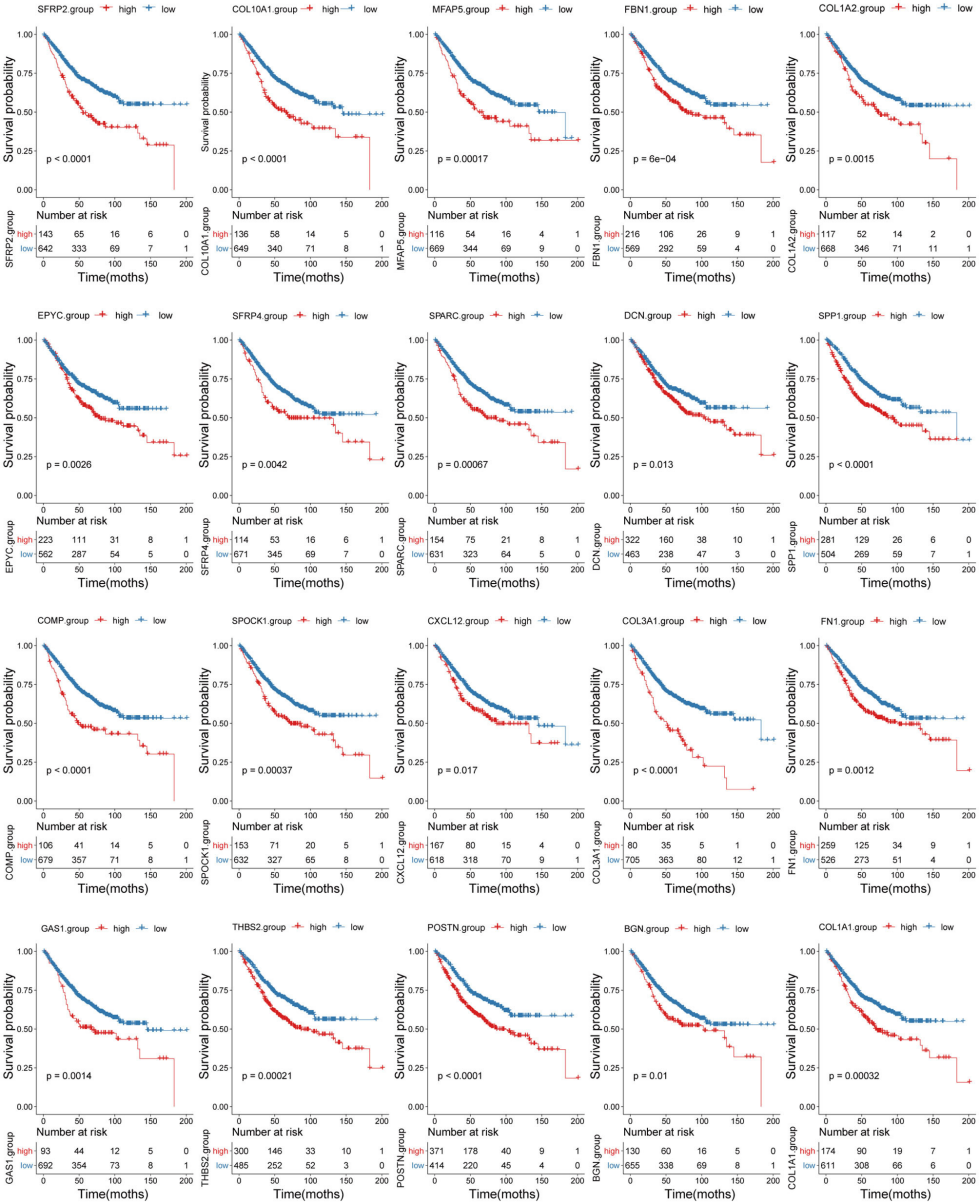
Correlation between the 20 biomarkers and survival prognosis. Patient survival curves based on the levels of each biomarker are shown.

Survival analyses using the training set showed that when applied separately, each candidate biomarker generated a significant survival difference between the high and low score groups (Figure 9). However, only 14/20 were validated in the testing set based on a threshold of *p* < 0.05 (Supplementary Figure 7). Two more were marginally significant [microfibril associated protein 5 (*p* = 0.056) and thrombospondin 2 (*p* = 0.051)] were retained. Therefore, we finally identified 16 tumor stroma biomarkers in this study, this suggests that these genes are closely related to the tumor stromal function and survival prognosis of CC patients.

### Identification of a New Prognosis Indicator for Risk Stratification

**Figure 10:**
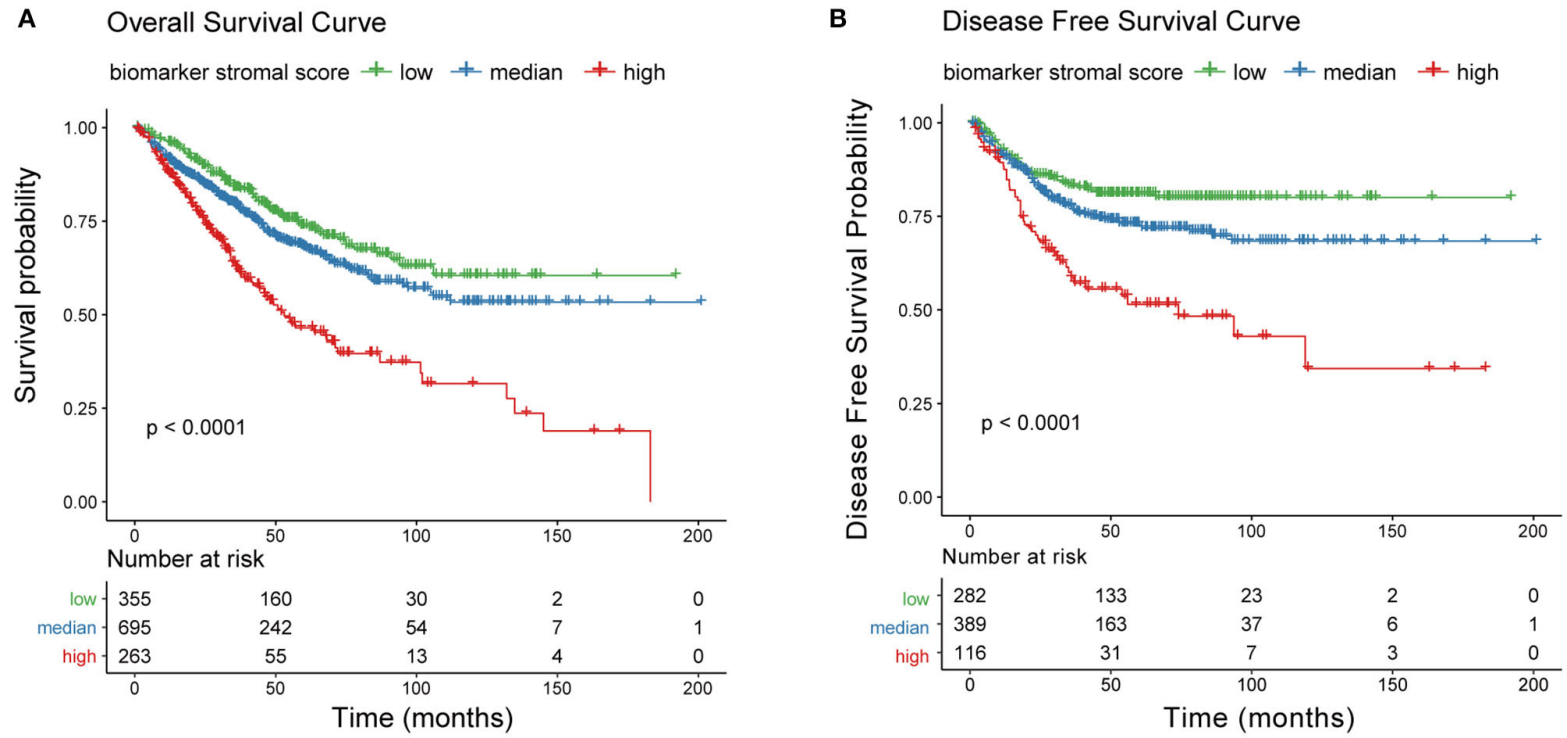
Correlation between the new prognosis indicator and survival prognosis. **(A)** Overall survival and **(B)** disease-free survival curves for three risk groups based on biomarker stromal score.

**Figure 11:**
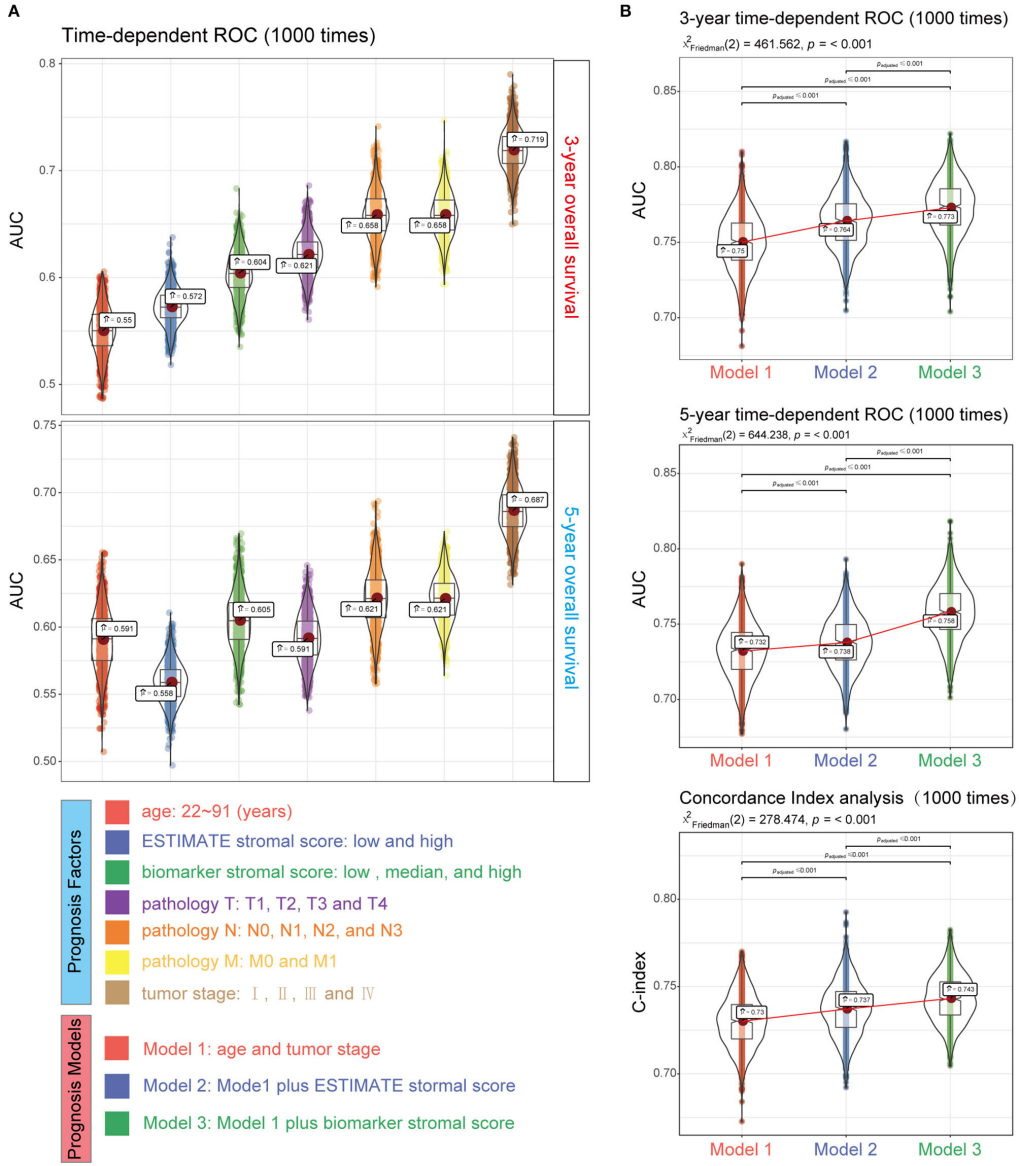
Performance of the biomarker stromal score in prognosis prediction compared with clinicopathological features. **(A)** Boxplots show the prediction accuracy for 3- and 5-year overall survival, based on the AUC with 1,000× bootstrap resampling for each parameter. **(B)** The top and middle boxplots show the prediction accuracy for 3- and 5- year overall survival based on the AUC with 1,000× bootstrap resampling, while the bottom boxplot shows the prediction accuracy for overall survival based on the C-index with 1,000× bootstrap resampling for each prognosis model.

We next generated a new prognosis indicator based on the 16 tumor stroma biomarkers, termed the biomarker stromal score, which ranged from 0 to 16. We divided 1,313 patients (all with complete OS information; 787 also had complete DFS information) into three risk groups based on thresholds of 0, 1–9, and 10–16. OS and DFS analyses both revealed significant survival differences between the three risk groups (Figures 10A,B). Time-dependent ROC analyses showed that the ability of the biomarker stromal score to predict 3- and 5-year OS was superior to the features of patient age and ESTIMATE stromal score and had similar AUC values to the T, N, and M pathology results. The tumor stage had the best prediction accuracy (Figure 11A). Therefore, the biomarker stromal score is a comparably effective prognosis indicator to known clinical features.

### Construction of the Prognosis Model

**Figure 12:**
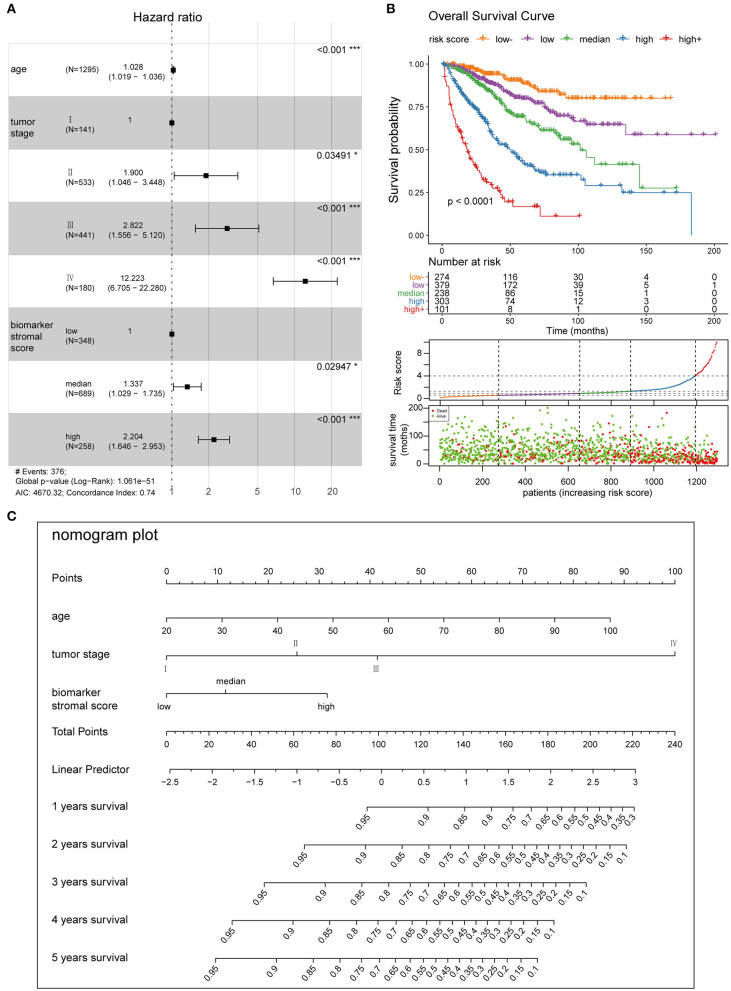
Clinical application of the best multivariable hazards model. **(A)** Forest plot of hazard ratios for the three prognosis features in model 3. **(B)** Survival curves and scatter plots of patients in five different risk groups, based on the risk score. **(C)** A nomogram plot was constructed with the three prognosis features to predict the 1–5-year overall survival rates of patients with CC.

We used four prognosis factors (age, tumor stage, the ESTIMATE stromal score, and the biomarker stromal score) in different combinations to construct prognosis models, and data on 1,295 patients with complete age, tumor stage, ESTIMATE stromal score, and biomarker stromal score information were used in multivariable regression analyses. Three prognosis models were constructed: model 1 included age and tumor stage; model 2 included age, tumor stage, and the ESTIMATE stromal score; and model 3 included age, tumor stage, and the biomarker stromal score. Time-dependent ROC (3- and 5-year) and C-index results revealed that model 3 had the best prediction accuracy (Figure 11B). The hazard ratios of each feature in model 3 are shown in Figure 12A. Model 3 risk scores ranged from 0.104 to 12.539, and patients were divided into five risk groups based on thresholds of ≤0.556, 0.557–0.896, 0.897–1.27, 1.28–3.99, and >3.99. Significant survival differences were observed between the five groups (Figure 12B). In the nomogram plot, weighted scores calculated based on the age, tumor stage, and biomarker stromal score were used to predict the 1–5-year OS rate of patients with CC (Figure 12C). The calibration curve demonstrated good performance for the nomogram plot compared to an ideal model (Supplementary Figure 8). Therefore, our findings suggest that the biomarker stromal score can improve CC survival prognosis prediction accuracy.

### Construction of Biomarker Regulatory Networks

**Figure 13:**
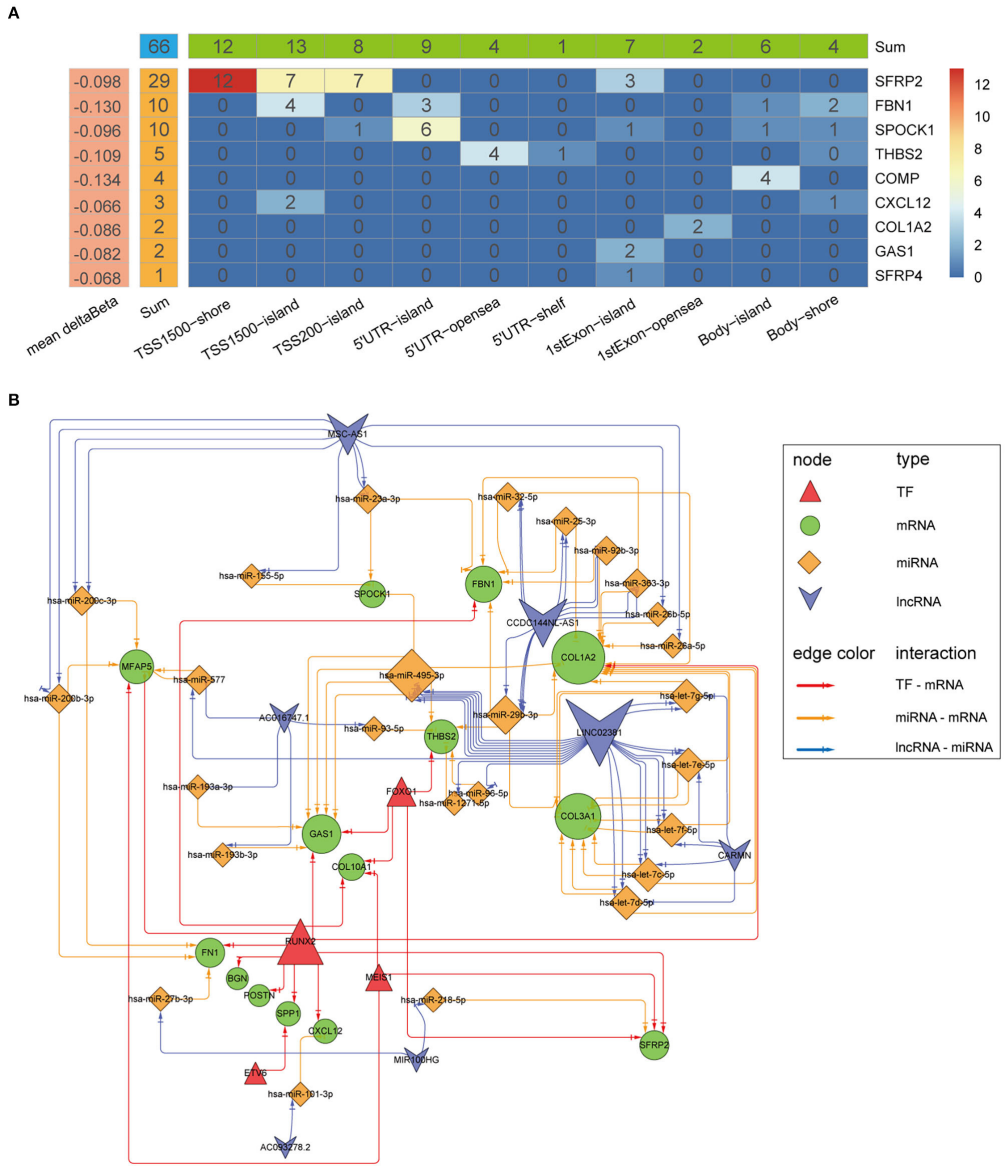
Mechanistic insight into the tumor stroma biomarkers. **(A)** Heatmap of demethylation site distribution in nine biomarkers. The x-axis indicates the region relative to the genome and CpG islands, and the numbers indicate the demethylation probe counts in the region. **(B)** A network of TF-mRNA and lncRNA-miRNA-mRNA interactions. The node size indicates the node edge count, and arrows represent direct regulatory effects.

Differential analysis of the methylation beta matrix between the high and low stromal score groups revealed that 9/16 biomarkers contained at least one significantly demethylated CpG site (a total of 66 CpG probes based on thresholds of deltaBeta < -0.05 and adj*P* < 0.05; Figure 13A and Supplementary Table 3). Among them, secreted frizzled related protein 2 (*SFRP2*) had the most demethylated sites (29; with a mean deltaBeta of −0.098). This suggests that increased demethylation contributes to the high expression of the biomarkers in the high stromal score group. Besides, survival analyses showed that 15/66 probes could make significant survival differences based on the optimal cutoff of each probes (*p* < 0.05; Supplementary Table 3). We next constructed a TF-mRNA regulatory network consisting of 4 TFs, 12 mRNAs, and a total of 19 edges; the interaction details are shown in Figure 13B and Supplementary Table 4. Interestingly, RUNX family transcription factor 2 (RUNX2) could regulate 11/12 mRNAs in the network. We also constructed a ceRNA network consisting of 7 lncRNAs, 26 miRNAs, and 10 mRNAs, with a total of 53 edges (Figure 13B and Supplementary Table 5). Survival analyses based on the lncRNAs and miRNAs included in the networks are shown in Supplementary Figures 9A,B. In summary, these regulatory networks provide new insights into the mechanism of tumor stroma biomarkers of CC.

## Discussion

Presently, risk stratification and prognosis prediction for patients with CC is mainly based on clinical and pathological characteristics (3, 4). In a recent study, Pagès et al. (12) demonstrated that a new indicator, the immunoscore, can effectively improve the accuracy of prognosis prediction for patients with CC. In this study, we have identified 16 tumor stroma biomarkers for primary CC and created a new indicator for risk stratification and prognosis prediction based on them. Our findings indicate that the tumor stroma is significantly negatively associated with survival prognosis, and that our new tumor stroma indicator could significantly improve the OS prediction accuracy of the currently used classification system.

It is well-known that interactions between cancer cells and the TME play important roles in tumor progression and therapeutic resistance (18, 19). While tumor cells have historically been the main therapeutic target of cancer treatment, different components of the TME, such as immune cells and angiogenic factors, have been recently targeted as well (20–23). However, these studies took limited notice of stromal components, and acquiring further insight into the interactions between cancer cells and the tumor stroma may provide novel biomarkers for stroma-targeted therapies as well as an increased understanding of drug resistance. Furthermore, there remains a lack of uniform criteria to assess tumor stroma condition. In this study, we assessed the CC tumor stroma by assigning scores based on stromal signatures generated using the ESTIMATE algorithm (13), and found that patients with high stromal scores had worse survival prognosis than patients with low stromal scores. Our findings in CC are consistent with results for several other cancers, such as gastric cancer, prostate cancer, and early-stage non-small cell lung cancer (14, 24–26). This indicates that the scores generated by this method may be a good tool to assess the CC tumor stroma condition and could be used as a prognosis factor for CC. In addition, we also found that the stroma score was significantly negatively correlated with the survival prognosis of chemotherapy patients, which may be caused by the resistance of tumor stroma to chemotherapy. Previous studies (27, 28) showed tumor-stromal architecture has been associated with modulation of the response to anti-angiogenic therapy, and combined therapy of chemotherapy and anti-angiogenesis was more effective than monotherapy. Therefore, the role of tumor stroma on anti-angiogenic therapy deserves further study.

While the notion that therapies targeting cancer cells and the TME are equally important is widely accepted (29), specific biomarkers of the tumor stroma are still lacking, and the molecular mechanisms by which the stroma affects the tumor remain unclear, because of its heterogeneity and complexity (30, 31). In this study, to clarify the biological processes and signaling pathways affected by the tumor stroma in the promotion of CC progression and chemotherapy resistance, we conducted enrichment analysis on the tumor stroma-related genes. Interestingly, in GO and KEGG analyses, the most statistically significant terms and pathways were related to the ECM: the BP “ECM organization” (adj*P* = 5.22E-59) and the KEGG pathway “ECM-receptor interaction” (adj*P* = 6.47E-11), respectively. Tumor progression results in ECM component changes and remodeling. This makes the ECM more conducive to promoting the growth, survival, and migration of cancer cells (32), and can increase drug resistance in various ways. For instance, the buildup of a rigid ECM surrounding tumor cells creates a physical barrier that reduces the diffusion of therapeutic agents (33, 34). Cancer cells can also evade chemotherapy by strongly adhering to ECM proteins through a process known as cell adhesion-mediated drug resistance (35–37). Our findings suggest that the ECM plays an important role in the progression and therapeutic resistance of CC. Two proven key signaling pathways related to tumor progression and chemotherapy resistance, the phosphatidylinositol 3-kinase (PI3K)-AKT serine/threonine kinase 1 (AKT1) and transforming growth factor β1 pathways (38–40), were also significantly enriched in our study. Most of the genes enriched in these two pathways were highly expressed in the high stromal score group. Our results therefore identify major biological processes and key signaling pathways related to the effects of the tumor stroma on CC, providing valuable clues for its treatment.

We identified 16 tumor stroma biomarkers that were closely related to the survival prognosis of patients with CC, and some have previously reported associations with CC tumor progression. For instance, fibronectin 1 (FN1) had the highest degree of connectivity in the PPI network. Xie et al. (41) showed that inhibiting FN1-SRC proto-oncogene, non-receptor tyrosine kinase/protein tyrosine kinase 2-guanosine triphosphatase (GTPase) signaling could inhibit CC metastasis, and Cai et al. (42) reported that FN1 depletion could inhibit colorectal carcinogenesis by suppressing proliferation, migration, and invasion. The significant DEGs *SFRP2* and *SFRP4*, and especially *SFRP2*, had the most demethylated sites and the biggest logFC values in our study, and are involved in the biological processes of “extracellular matrix organization” and “extracellular structure organization.” Vincent et al. (43) reported that *SFRP2* and *SFRP4* are typically associated with poor prognosis concomitant with epithelial-to-mesenchymal transition (EMT). Nfonsam et al. (44) found that patients with CC that overexpress SFRP4 have poor OS. In these patients, SFRP4 levels were negatively correlated with the levels of the EMT suppressors claudin 4 (CLDN4), claudin 7 (CLDN7), tight junction protein 3 (TJP3), mucin 1, cell surface associated (MUC1), and cadherin 1 (CDH1). Klement et al. (45) demonstrated that high SPP1 expression was associated with decreased OS by acting as an immune checkpoint to suppress T cell activation. C-X-C motif chemokine ligand 12 (CXCL12), secreted by fibroblasts, can promote the proliferation and invasion of CC via the PTEN/PI3K/Akt and MAPK/PI3K/AP-1 signaling pathways (46–48). In addition, its receptor C-X-C motif chemokine receptor 4 (CXCR4) has been used as an effective therapeutic target in prostate cancer (49–51). Thus, the findings of these studies further support our results.

Regarding the regulatory mechanisms of the biomarkers, we were surprised to find that RUNX2 could regulate 11/12 mRNAs in the TF-mRNA network. Increasing evidence has highlighted the importance of RUNX2 in a variety of cancers. For instance, it is highly expressed in metastatic prostate cancer cells and may play an important role in prostate cancer-derived metastatic bone disease (52, 53). RUNX2 plays an oncogenic role in esophageal carcinoma by activating the PI3K/AKT1 and extracellular-regulated kinase signaling pathways (54). Targeting RUNX2 represses cell growth and metastasis in lung cancer cells (55) and inhibits the progression of breast cancer to metastatic bone disease (56). Besides, regarding the regulatory function of RUNX2 in the network, Francisco et al. (57) reported elevated RUNX2 may transcriptionally activate genes mediating osteosarcoma progression and metastasis by targeting SPP1. Toshihisa el al. (58) reported that Runx2 could induce the expression of major bone matrix protein genes, including COL1A1, SPP1, and FN1, *in vitro*. Besides, Toshihisa el al. (59) also reported Runx2 plays an important role in the bone metastasis of breast and prostate cancers by up-regulating SPP1. Although some regulatory relationships in the network have been verified by previous studies, there are still many waiting for further verification. However, despite increasing evidence of the importance of RUNX2 in various cancers, there are no reports about its relevance in CC. Our results suggest that the role of RUNX2 in CC is worthy of further study.

The current risk classification for cancers is mainly based on the TNM staging system (3); however, for a deeper understanding of tumor progression, more prognosis factors should be considered. For instance, Weiser et al. showed that an extended prognosis model including TNM staging, the tumor grade, the number of collected metastatic lymph nodes, age, and sex had higher sensitivity and specificity for CC (the C-index rose from 0.60 to 0.68) than a model using the TNM system alone (4). Pagès et al. (12) showed that adding an immunoscore to a model combining clinical variables can significantly improve OS prediction accuracy of AUC from 0.6 to 0.62. In this study, we created a new prognosis indicator based on tumor stroma biomarkers. Adding this indicator to a prognosis model based on age and tumor stage also significantly improved the prediction accuracy, with a similar degree of improvement to Pagès's immunoscore (3-year AUC raised from 0.75 to 0.773; 5-year AUC raised from 0.732 to 0.758). In addition, as the new indicator is based on only 16 biomarkers, testing will be easier, more effective, and more economically feasible for patients with CC vs. the ESTIMATE stromal score, which is based on 141 signatures.

Our study demonstrates the important role of the tumor stroma in CC tumor progression and chemotherapy resistance and provides novel candidates for targeted CC therapies. However, the data available for this study is limited, and our findings are mainly obtained through bioinformatics analysis of high-throughput data which have inevitable batch differences between different datasets due to sequencing technologies, so these findings will require further validation with more clinical data and molecular experiments. In our future work, we will test additional clinical datasets and perform additional molecular experimental verification on the identified biomarkers. Notable, this is the first study to consider the tumor stroma in CC risk stratification, and the new prognosis indicator and prognosis model created in this study will increase the accuracy of risk stratification and survival prediction, improving the outcomes of patients with CC.

## Data Availability Statement

The original contributions presented in the study are included in the article/Supplementary Materials, further inquiries can be directed to the corresponding author/s.

## Author Contributions

This study was performed in collaboration among all authors. YC and XL contributed to the study design. YC, WW, and BJ downloaded the datasets and performed the statistical analyses. LY and FX analyzed the results. YC drafted the manuscript, and all authors contributed to revision of the final manuscript. LY supervised the study and manuscript preparation.

## Conflict of Interest

The authors declare that the research was conducted in the absence of any commercial or financial relationships that could be construed as a potential conflict of interest.
