# Supplementary material for: Integrating Tumor Stroma Biomarkers With Clinical Indicators for Colon Cancer Survival Stratification
Source: Front Med (Lausanne). 2020 Dec 7;7:584747. doi: 10.3389/fmed.2020.584747 (PMC7750539; doi:10.3389/fmed.2020.584747)
Supplement: Supplementary file 1 [file Table_1.DOCX]

| Characters | Dataset785 | Dataset428 | Dataset100 |
| --- | --- | --- | --- |
| age (mean/years) | 66.13 | 66.67 | 65.43 |
| gender |  |  |  |
| male | 427 | 197 | 58 |
| female | 358 | 231 | 42 |
| pathology T |  |  |  |
| T1 | 11 | 10 | 0 |
| T2 | 44 | 75 | 13 |
| T3 | 360 | 293 | 74 |
| T4 | 117 | 49 | 13 |
| pathology N |  |  |  |
| N0 | 295 | 251 | 53 |
| N1 | 131 | 101 | 32 |
| N2 | 98 | 76 | 13 |
| N3 | 6 | 0 | 0 |
| pathology M |  |  |  |
| M0 | 474 | 317 | 93 |
| M1 | 60 | 59 | 7 |
| tumor stage |  |  |  |
| Ⅰ | 60 | 72 | 10 |
| Ⅱ | 329 | 164 | 41 |
| Ⅲ | 278 | 122 | 42 |
| Ⅳ | 114 | 59 | 7 |
| ESTIMATE stromal score |  |  |  |
| low | 560 | 348 | 75 |
| high | 225 | 80 | 25 |
| biomarker stromal score |  |  |  |
| low | 287 | 42 | 26 |
| median | 388 | 261 | 46 |
| high | 110 | 125 | 28 |
| living status |  |  |  |
| dead | 278 | 332 | 92 |
| alive | 507 | 96 | 8 |
| DFS status |  |  |  |
| Yes | 187 | - | 7 |
| No | 516 | - | 77 |

Supplementary Table 1. Clinical statistics of patients included in this study

A total of 1313 patients with primary colon cancer were included in this study. DFS, disease free survival.
