# Supplementary material for: Integrating Tumor Stroma Biomarkers With Clinical Indicators for Colon Cancer Survival Stratification
Source: Front Med (Lausanne). 2020 Dec 7;7:584747. doi: 10.3389/fmed.2020.584747 (PMC7750539; doi:10.3389/fmed.2020.584747)
Supplement: Supplementary file 2 [file Table_2.DOCX]

Supplementary Table 2. Expression difference of the biomarkers at protein/phosphorylation level

| protein/phosphorylation | highMean | lowMean | DE.value | pValue |
| --- | --- | --- | --- | --- |
| BGN\|BGN | 0.77 | -0.25 | 1.03 | 1.6E-07 |
| COL10A1\|COL10A1 | 0.77 | -0.28 | 1.05 | 3.1E-08 |
| COL1A1\|COL1A1 | 0.73 | -0.25 | 0.98 | 1.2E-07 |
| COL1A1\|COL1A1_pS176 | 0.82 | -0.28 | 1.1 | 2.7E-06 |
| COL1A2\|COL1A2 | 0.72 | -0.24 | 0.96 | 2.9E-07 |
| COL3A1\|COL3A1 | 0.76 | -0.26 | 1.01 | 1.2E-07 |
| COMP\|COMP | 0.77 | -0.25 | 1.02 | 4.5E-06 |
| CXCL12\|CXCL12 | 0.54 | -0.18 | 0.73 | 1.5E-03 |
| DCN\|DCN | 0.74 | -0.25 | 0.99 | 1.2E-06 |
| FBN1\|FBN1 | 0.51 | -0.16 | 0.67 | 6.4E-04 |
| FBN1\|FBN1_pS2702 | 0.55 | -0.18 | 0.73 | 1.1E-03 |
| FBN1\|FBN1_pS2709 | 0.53 | -0.18 | 0.71 | 3.0E-03 |
| FN1\|FN1 | 0.6 | -0.2 | 0.8 | 4.2E-04 |
| FN1\|FN1_pS2384 | 0.46 | -0.14 | 0.61 | 5.6E-03 |
| MFAP5\|MFAP5 | 0.67 | -0.22 | 0.89 | 1.3E-04 |
| POSTN\|POSTN | 0.73 | -0.24 | 0.96 | 8.4E-07 |
| SFRP2\|SFRP2 | 0.69 | -0.23 | 0.91 | 2.4E-05 |
| SFRP4\|SFRP4 | 0.48 | -0.17 | 0.65 | 5.0E-03 |
| SPARC\|SPARC | 0.66 | -0.22 | 0.88 | 9.7E-05 |
| SPP1\|SPP1_pS219 | 0.79 | -0.27 | 1.06 | 1.5E-04 |
| SPP1\|SPP1_pS234 | 0.69 | -0.33 | 1.02 | 2.8E-05 |
| SPP1\|SPP1_pS254 | 0.59 | -0.28 | 0.87 | 1.2E-04 |
| THBS2\|THBS2 | 0.68 | -0.23 | 0.91 | 1.1E-04 |

HighMean, the average expression level of protein/phosphorylation in the high ESTIMATE stromal score group; LowMean, the average expression level of protein/phosphorylation in the low ESTIMATE stromal score group; DE.value, equal to highMean minus lowMean; pValue was caculated by T-test between the high- and low- ESTIMATE score groups.
