# Supplementary material for: Integrating Tumor Stroma Biomarkers With Clinical Indicators for Colon Cancer Survival Stratification
Source: Front Med (Lausanne). 2020 Dec 7;7:584747. doi: 10.3389/fmed.2020.584747 (PMC7750539; doi:10.3389/fmed.2020.584747)
Supplement: Supplementary file 4 [file Table_4.DOCX]

Supplementary Table 4. The interaction details of the TF-mRNA network

| TF\| phosphorylation | Gene | Cor | P.value（cor） | HighMean | LowMean | DE.value | P.value（DE） |
| --- | --- | --- | --- | --- | --- | --- | --- |
| ETV6\|ETV6_pT18 | SPP1 | 0.399 | 9.90E-03 | 0.601 | -0.198 | 0.799 | 2.30E-03 |
| FOXO1\|FOXO1_pS287 | COL10A1 | 0.379 | 2.40E-02 | 0.717 | -0.202 | 0.919 | 2.80E-05 |
| FOXO1\|FOXO1_pS287 | GAS1 | 0.461 | 4.20E-04 | 0.717 | -0.202 | 0.919 | 2.80E-05 |
| FOXO1\|FOXO1_pS287 | SFRP2 | 0.419 | 3.90E-03 | 0.717 | -0.202 | 0.919 | 2.80E-05 |
| FOXO1\|FOXO1_pS287 | THBS2 | 0.369 | 3.60E-02 | 0.717 | -0.202 | 0.919 | 2.80E-05 |
| MEIS1\|MEIS1_pS196 | COL10A1 | 0.402 | 8.50E-03 | 0.541 | -0.184 | 0.725 | 5.90E-04 |
| MEIS1\|MEIS1_pS196 | MFAP5 | 0.396 | 1.10E-02 | 0.541 | -0.184 | 0.725 | 5.90E-04 |
| MEIS1\|MEIS1_pS196 | SFRP2 | 0.405 | 7.60E-03 | 0.541 | -0.184 | 0.725 | 5.90E-04 |
| RUNX2\|RUNX2_pS28 | BGN | 0.495 | 5.40E-05 | 0.641 | -0.261 | 0.902 | 1.90E-04 |
| RUNX2\|RUNX2_pS28 | COL10A1 | 0.656 | 7.70E-11 | 0.641 | -0.261 | 0.902 | 1.90E-04 |
| RUNX2\|RUNX2_pS28 | COL1A2 | 0.412 | 5.40E-03 | 0.641 | -0.261 | 0.902 | 1.90E-04 |
| RUNX2\|RUNX2_pS28 | CXCL12 | 0.483 | 1.20E-04 | 0.641 | -0.261 | 0.902 | 1.90E-04 |
| RUNX2\|RUNX2_pS28 | FBN1 | 0.475 | 1.90E-04 | 0.641 | -0.261 | 0.902 | 1.90E-04 |
| RUNX2\|RUNX2_pS28 | FN1 | 0.515 | 1.50E-05 | 0.641 | -0.261 | 0.902 | 1.90E-04 |
| RUNX2\|RUNX2_pS28 | GAS1 | 0.601 | 1.90E-08 | 0.641 | -0.261 | 0.902 | 1.90E-04 |
| RUNX2\|RUNX2_pS28 | MFAP5 | 0.563 | 4.40E-07 | 0.641 | -0.261 | 0.902 | 1.90E-04 |
| RUNX2\|RUNX2_pS28 | POSTN | 0.51 | 2.00E-05 | 0.641 | -0.261 | 0.902 | 1.90E-04 |
| RUNX2\|RUNX2_pS28 | SFRP2 | 0.591 | 4.60E-08 | 0.641 | -0.261 | 0.902 | 1.90E-04 |
| RUNX2\|RUNX2_pS28 | SPP1 | 0.592 | 4.00E-08 | 0.641 | -0.261 | 0.902 | 1.90E-04 |

TF, transcription factor; Cor, indicates the relationship between the TF and mRNA calculated by Pearson analysis; HighMean, the average phosphorylation level of TF in the high ESTIMATE stromal score group; LowMean, the average phosphorylation level of TF in the low ESTIMATE stromal score group; DE.value, differentially expressed value, equal to highMean minus lowMean.
