# Supplementary material for: Integrating Tumor Stroma Biomarkers With Clinical Indicators for Colon Cancer Survival Stratification
Source: Front Med (Lausanne). 2020 Dec 7;7:584747. doi: 10.3389/fmed.2020.584747 (PMC7750539; doi:10.3389/fmed.2020.584747)
Supplement: Supplementary file 5 [file Table_5.DOCX]

Supplementary Table 5. The interaction details of the lncRNA-miRNA-mRNA network

| lncRNA | miRNA | mRNA | cor | P.value |
| --- | --- | --- | --- | --- |
| AC016747.1 | hsa-miR-193a-3p | GAS1 | 0.677 | 3.8E-58 |
| AC016747.1 | hsa-miR-193b-3p | GAS1 | 0.677 | 3.8E-58 |
| AC016747.1 | hsa-miR-577 | MFAP5 | 0.714 | 5.3E-68 |
| AC016747.1 | hsa-miR-93-5p | THBS2 | 0.694 | 2.6E-62 |
| AC093278.2 | hsa-miR-101-3p | CXCL12 | 0.664 | 5.4E-55 |
| CARMN | hsa-let-7e-5p | COL3A1 | 0.653 | 1.6E-52 |
| CARMN | hsa-let-7c-5p | COL3A1 | 0.653 | 1.6E-52 |
| CARMN | hsa-let-7d-5p | COL3A1 | 0.653 | 1.6E-52 |
| CARMN | hsa-let-7g-5p | COL3A1 | 0.653 | 1.6E-52 |
| CARMN | hsa-let-7f-5p | COL3A1 | 0.653 | 1.6E-52 |
| CCDC144NL-AS1 | hsa-miR-29b-3p | COL1A2 | 0.682 | 2.4E-59 |
| CCDC144NL-AS1 | hsa-miR-32-5p | COL1A2 | 0.682 | 2.4E-59 |
| CCDC144NL-AS1 | hsa-miR-363-3p | COL1A2 | 0.682 | 2.4E-59 |
| CCDC144NL-AS1 | hsa-miR-25-3p | COL1A2 | 0.682 | 2.4E-59 |
| CCDC144NL-AS1 | hsa-miR-92b-3p | COL1A2 | 0.682 | 2.4E-59 |
| CCDC144NL-AS1 | hsa-miR-29b-3p | COL3A1 | 0.688 | 9.3E-61 |
| CCDC144NL-AS1 | hsa-miR-29b-3p | FBN1 | 0.724 | 1.0E-70 |
| CCDC144NL-AS1 | hsa-miR-32-5p | FBN1 | 0.724 | 1.0E-70 |
| CCDC144NL-AS1 | hsa-miR-363-3p | FBN1 | 0.724 | 1.0E-70 |
| CCDC144NL-AS1 | hsa-miR-25-3p | FBN1 | 0.724 | 1.0E-70 |
| CCDC144NL-AS1 | hsa-miR-92b-3p | FBN1 | 0.724 | 1.0E-70 |
| CCDC144NL-AS1 | hsa-miR-29b-3p | THBS2 | 0.731 | 4.4E-73 |
| LINC02381 | hsa-miR-495-3p | COL1A2 | 0.659 | 6.4E-54 |
| LINC02381 | hsa-let-7g-5p | COL1A2 | 0.659 | 6.4E-54 |
| LINC02381 | hsa-let-7c-5p | COL1A2 | 0.659 | 6.4E-54 |
| LINC02381 | hsa-let-7d-5p | COL1A2 | 0.659 | 6.4E-54 |
| LINC02381 | hsa-let-7e-5p | COL1A2 | 0.659 | 6.4E-54 |
| LINC02381 | hsa-let-7f-5p | COL1A2 | 0.659 | 6.4E-54 |
| LINC02381 | hsa-let-7g-5p | COL3A1 | 0.683 | 1.8E-59 |
| LINC02381 | hsa-let-7c-5p | COL3A1 | 0.683 | 1.8E-59 |
| LINC02381 | hsa-let-7d-5p | COL3A1 | 0.683 | 1.8E-59 |
| LINC02381 | hsa-let-7e-5p | COL3A1 | 0.683 | 1.8E-59 |
| LINC02381 | hsa-let-7f-5p | COL3A1 | 0.683 | 1.8E-59 |
| LINC02381 | hsa-miR-495-3p | GAS1 | 0.651 | 3.9E-52 |
| LINC02381 | hsa-miR-495-3p | GAS1 | 0.651 | 3.9E-52 |
| LINC02381 | hsa-miR-495-3p | GAS1 | 0.651 | 3.9E-52 |
| LINC02381 | hsa-miR-495-3p | GAS1 | 0.651 | 3.9E-52 |
| LINC02381 | hsa-miR-577 | MFAP5 | 0.674 | 2.6E-57 |
| LINC02381 | hsa-miR-495-3p | SPOCK1 | 0.664 | 4.9E-55 |
| LINC02381 | hsa-miR-495-3p | THBS2 | 0.676 | 9.4E-58 |
| LINC02381 | hsa-miR-96-5p | THBS2 | 0.676 | 9.4E-58 |
| LINC02381 | hsa-miR-1271-5p | THBS2 | 0.676 | 9.4E-58 |
| MIR100HG | hsa-miR-27b-3p | FN1 | 0.657 | 1.9E-53 |
| MIR100HG | hsa-miR-218-5p | SFRP2 | 0.759 | 3.3E-82 |
| MSC-AS1 | hsa-miR-26a-5p | COL1A2 | 0.772 | 7.7E-87 |
| MSC-AS1 | hsa-miR-26b-5p | COL1A2 | 0.772 | 7.7E-87 |
| MSC-AS1 | hsa-miR-23a-3p | FBN1 | 0.839 | 1.7E-117 |
| MSC-AS1 | hsa-miR-200b-3p | FN1 | 0.692 | 7.5E-62 |
| MSC-AS1 | hsa-miR-200c-3p | FN1 | 0.692 | 7.5E-62 |
| MSC-AS1 | hsa-miR-200b-3p | MFAP5 | 0.742 | 2.2E-76 |
| MSC-AS1 | hsa-miR-200c-3p | MFAP5 | 0.742 | 2.2E-76 |
| MSC-AS1 | hsa-miR-155-5p | SPOCK1 | 0.781 | 2.9E-90 |
| MSC-AS1 | hsa-miR-23a-3p | SPOCK1 | 0.781 | 2.9E-90 |

Cor, indicates the relationship between lncRNA and mRNA calculated by Pearson analysis.
