# Supplementary material for: Integrating Tumor Stroma Biomarkers With Clinical Indicators for Colon Cancer Survival Stratification
Source: Front Med (Lausanne). 2020 Dec 7;7:584747. doi: 10.3389/fmed.2020.584747 (PMC7750539; doi:10.3389/fmed.2020.584747)
Supplement: Supplementary file 6 [file Data_Sheet_1.pdf]

Supplementary Figures

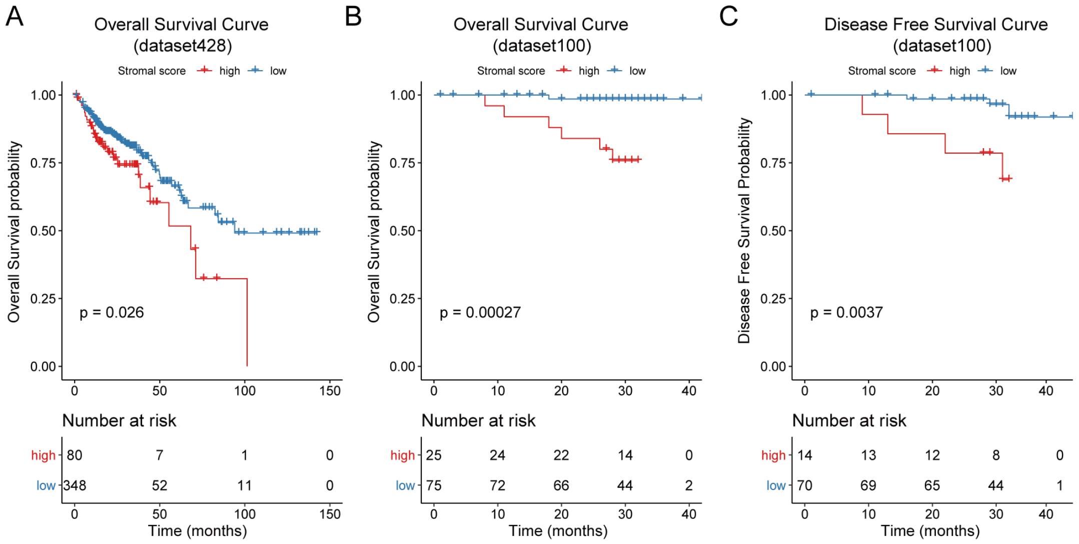

Supplementary Figure 1. Correlations between the ESTIMATE stromal score and CC survival prognosis were validated in testing sets of dataset428 **(A)** and dataset100 **(B-C)**.

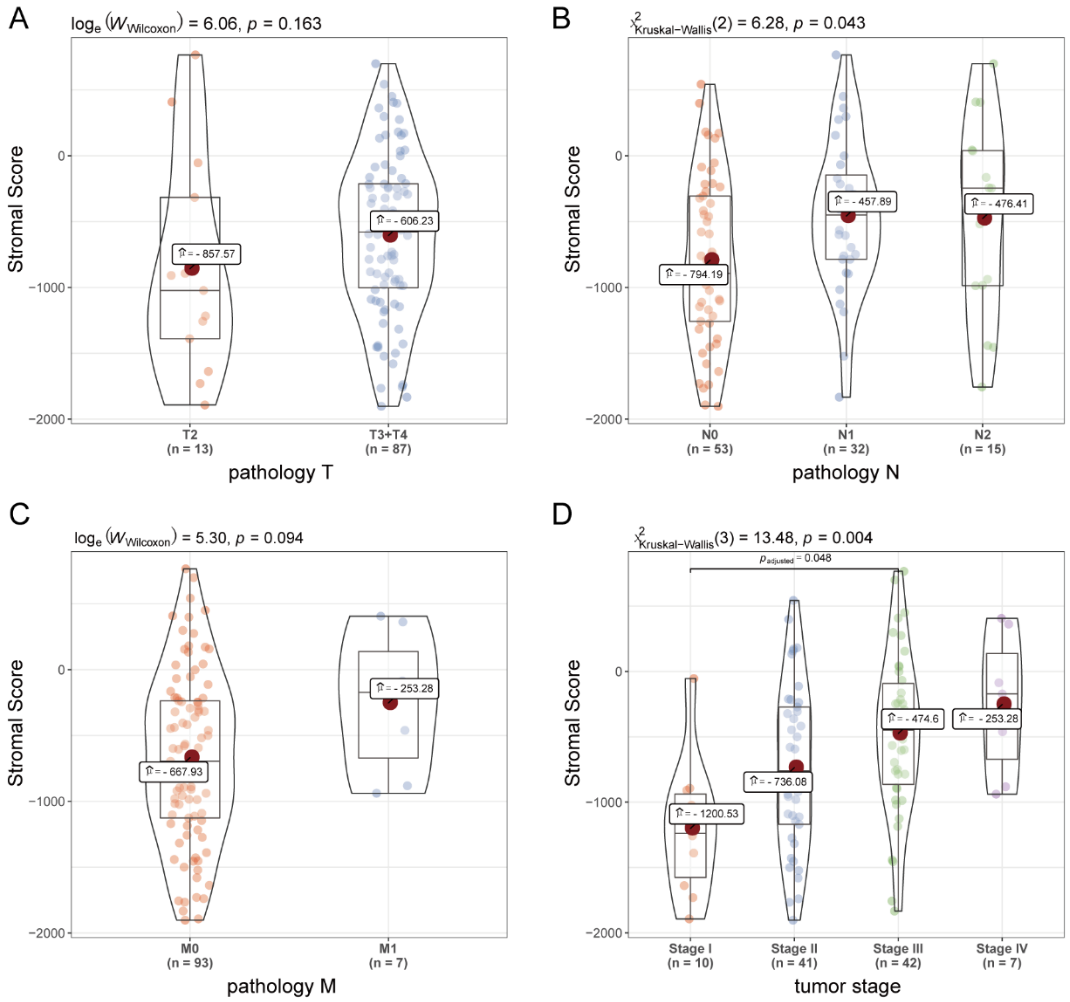

Supplementary Figure 2. Association between the ESTIMATE stromal score and clinical features of pathologic T **(A)**, pathologic N **(B)**, pathologic M **(C)**, tumor stage **(D)** were validated in the testing set of dataset100.

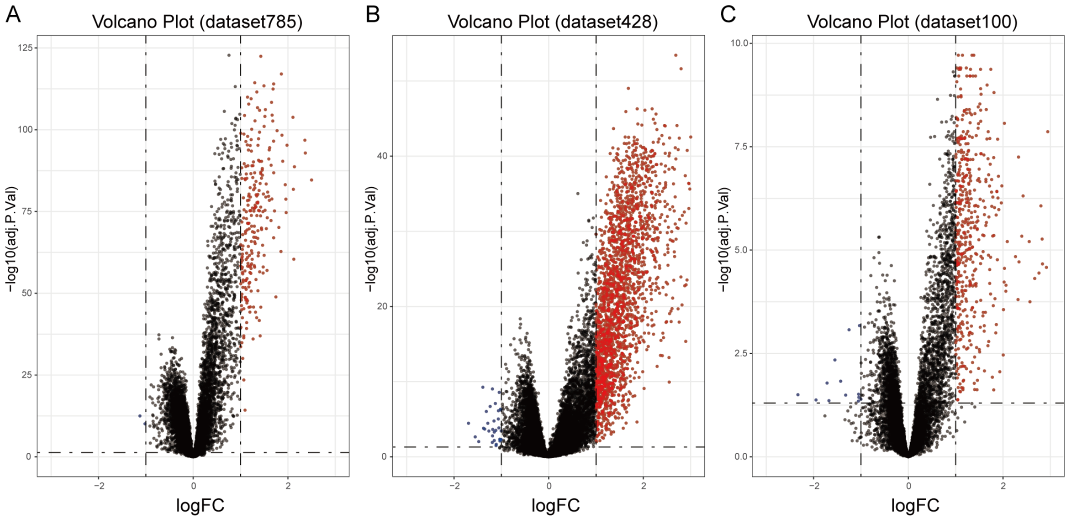

Supplementary Figure 3. Volcano plots of differential expressed genes between the high and low ESTIMATE stromal score groups. **(A)** 246 DEGs in dataset785. **(B)** 2313 DEGs in dataset428. **(C)** 501 DEGs in dataset100. Red and blue indicate up- and down-regulated genes, respectively.

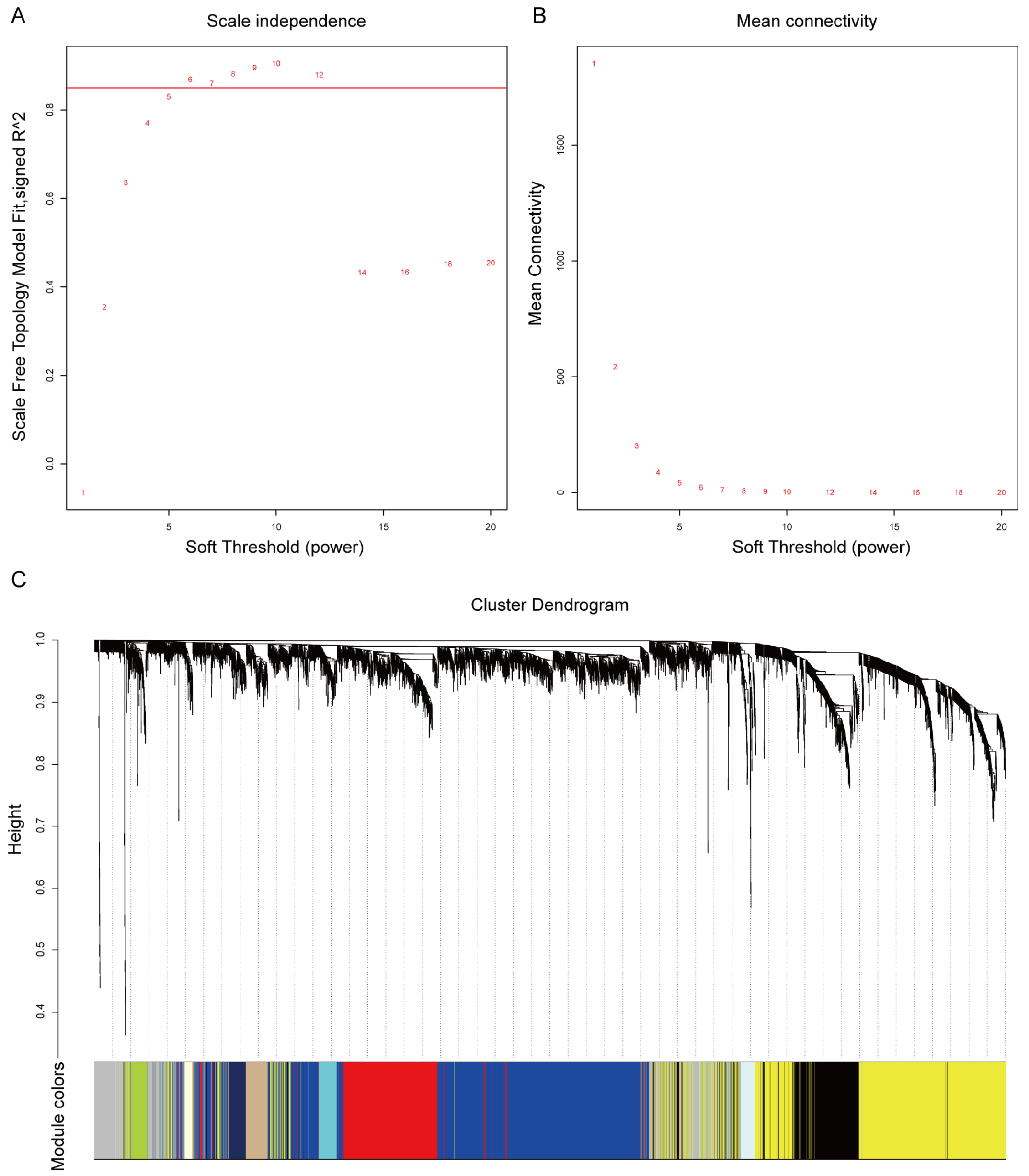

Supplementary Figure 4. Weighted gene co-expression network construction. **(A-B)** The scale-free fit index and the mean connectivity with various soft-thresholding powers, 6 was selected as the appropriate sort-thresholding power based on the cutoff of 0.85. **(C)** Clustering dendrograms of 21 mRNA modules.

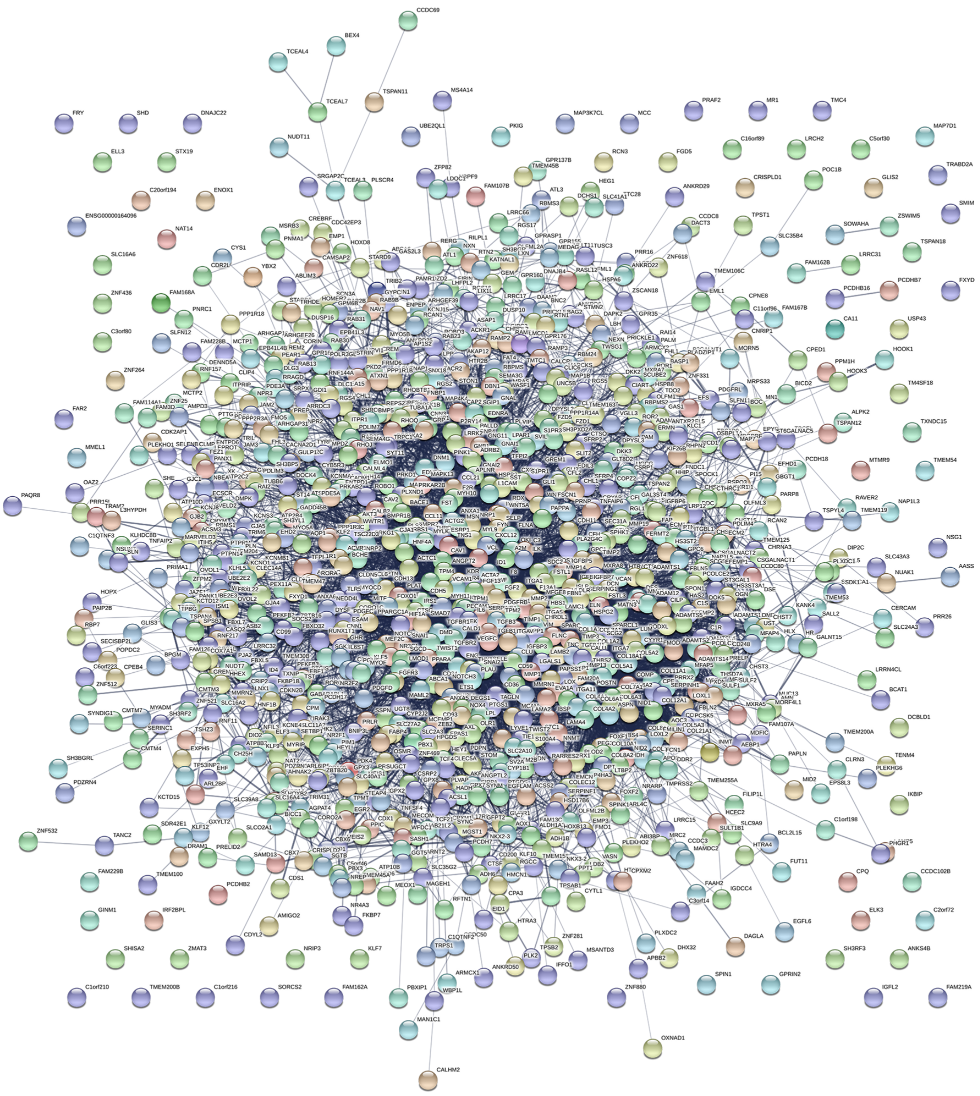

Supplementary Figure 5. The PPI network was constructed through the STRING database using the genes in the yellow module and contained a total of 1105 nodes and 8927 edges.

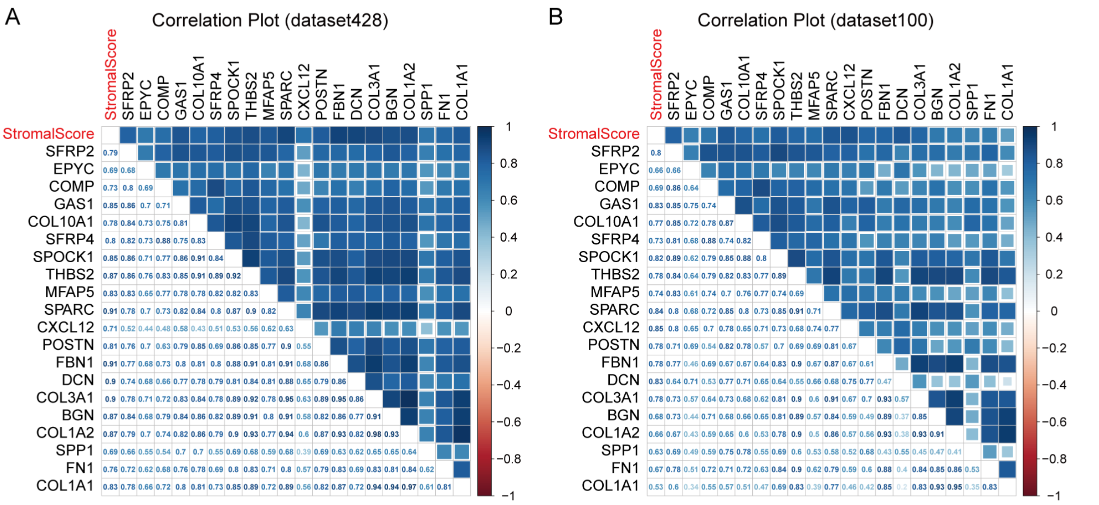

Supplementary Figure 6. Correlation between the ESTIMATE stromal score and the 20 candidate biomarkers of tumor stroma by Pearson correlation analysis were validated in the testing sets of dataset428 **(A)** and dataset100 **(B)**.

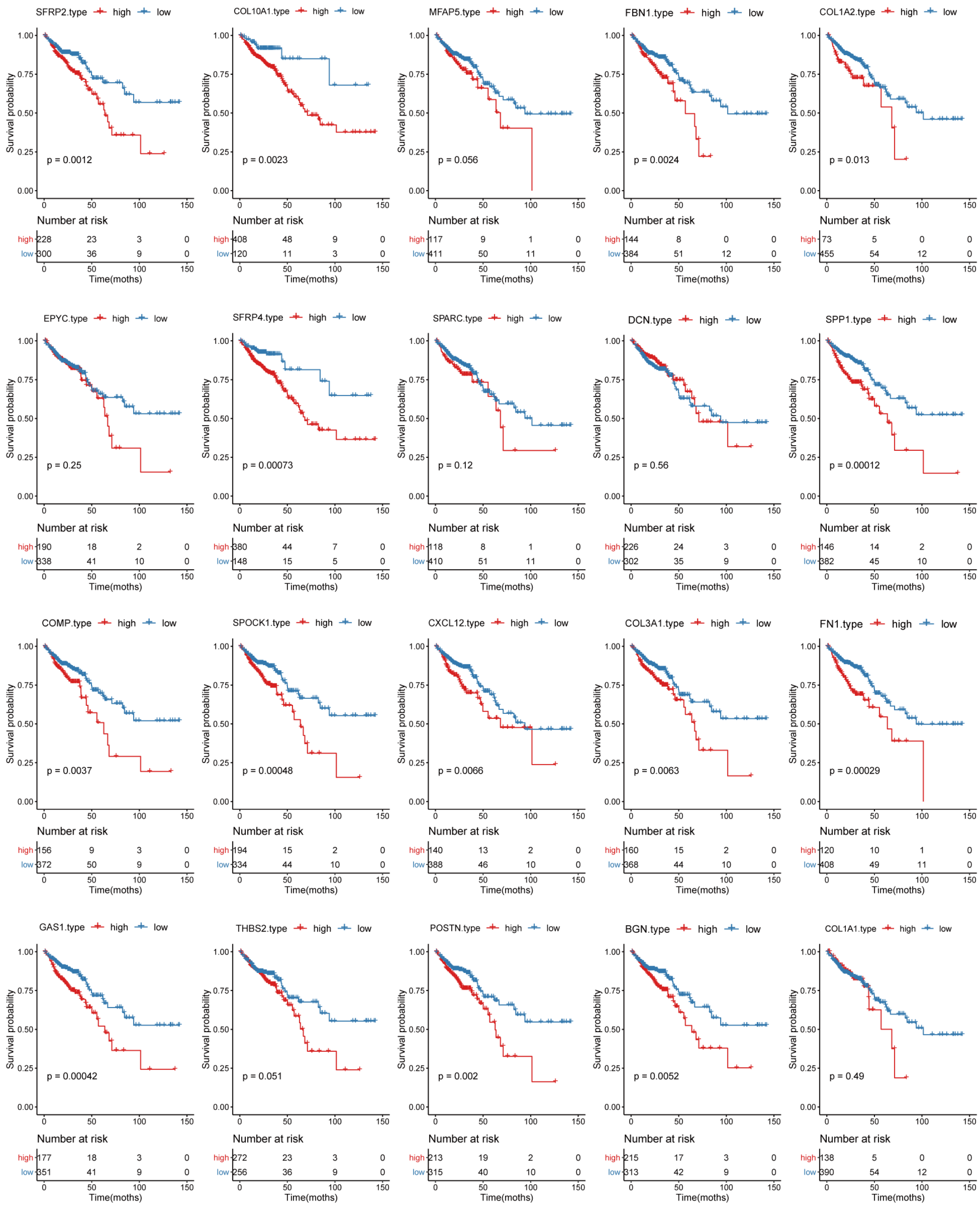

Supplementary Figure 7. A total of 528 patients from the testing datasets of dataset428 and dataset100 were used to validate the relationship of the 20 candidate biomarkers with survival prognosis. 4 candidate biomarkers (SPARC, DCN, COL1A1, EPYC) were not statistically significant and were filtered out.

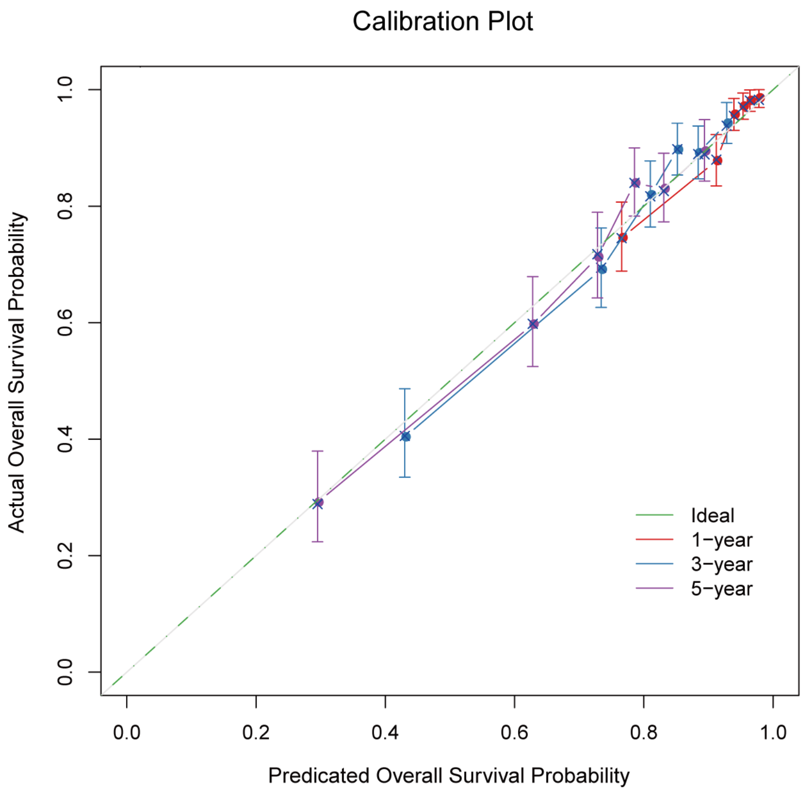

Supplementary Figure 8. The calibration plot showed the nomogram plot performed well compared against an ideal model for 1-, 3-, and 5-year overall survival prediction.

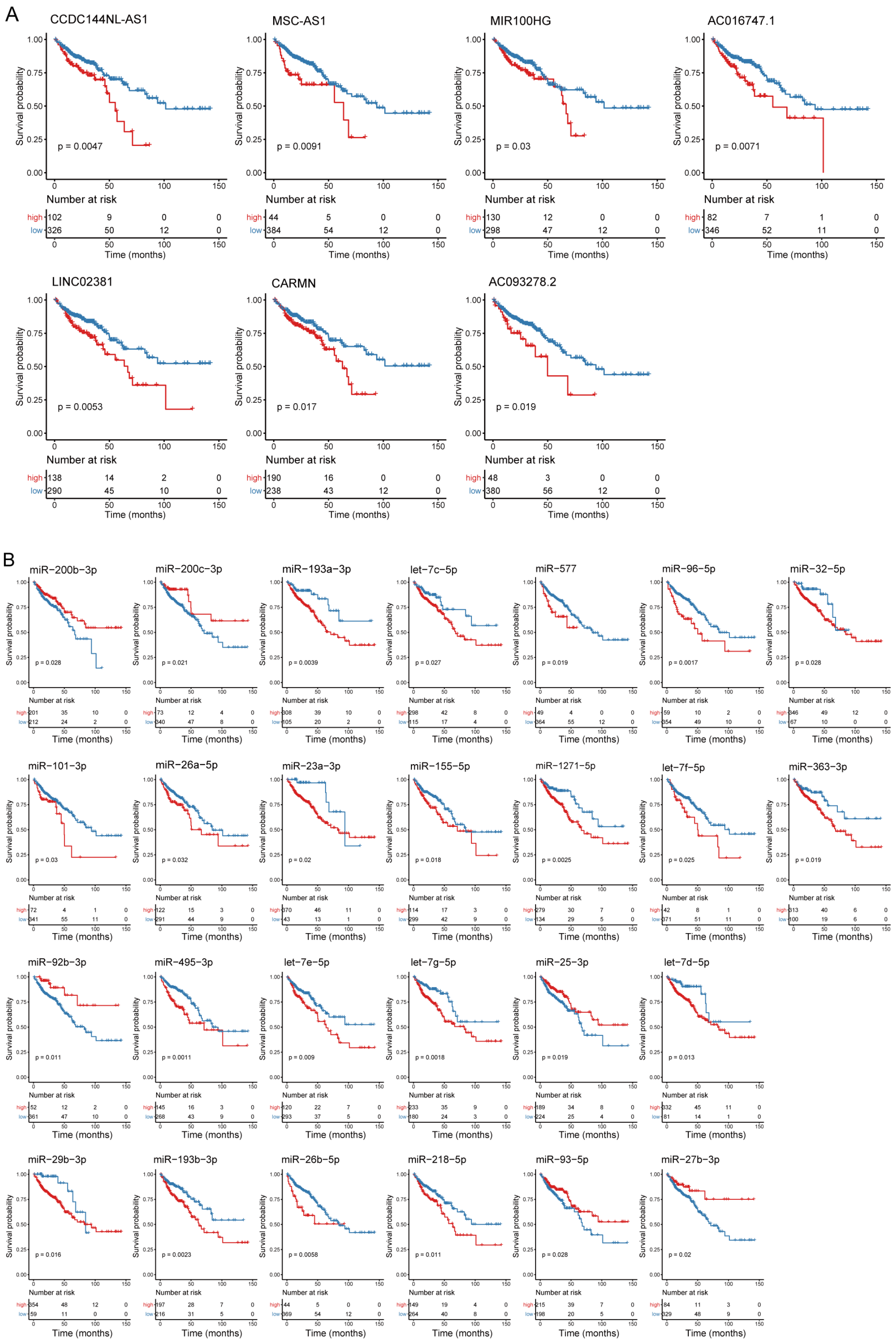

Supplementary Figure 9. Kaplan-Meier survival curves of 7 lncRNAs **(A)** and 26 miRNAs **(B)** in the ceRNA regulatory network. All these genes were statistically significant (p < 0.05).
